# The evolution of extreme cooperation via shared dysphoric experiences

**DOI:** 10.1038/srep44292

**Published:** 2017-03-14

**Authors:** Harvey Whitehouse, Jonathan Jong, Michael D. Buhrmester, Ángel Gómez, Brock Bastian, Christopher M. Kavanagh, Martha Newson, Miriam Matthews, Jonathan A. Lanman, Ryan McKay, Sergey Gavrilets

**Affiliations:** 1 Institute of Cognitive and Evolutionary Anthropology, University of Oxford, 51-53 Banbury Road, Oxford OX2 6PE UK; 2 Centre for Research in Psychology, Behaviour and Achievement, Coventry University, Priory Street, Coventry CV1 5FB, UK.; 3 Departamento de Psicología Social y de las Organizaciones Universidad Nacional de Educación a Distancia, UNED C/Juan del Rosal 10, Dcho, 1.58 Madrid, Spain; 4 Melbourne School of Psychological Sciences, The University of Melbourne, Australia; 5 RAND Corporation, 1200 South Hayes Street, Arlington, VA 22202-5050 USA; 6 Institute of Cognition and Culture, Queen’s University Belfast, 2-4 Fitzwilliam Street, Belfast, BT7 1NN UK; 7 ARC Centre of Excellence in Cognition and its Disorders, Department of Psychology, Royal Holloway, University of London, Egham, Surrey TW20 0EX, UK; 8 Department of Ecology and Evolutionary Biology, Department of Mathematics, National Institute for Mathematical and Biological Synthesis, University of Tennessee, Knoxville, TN 37996 USA

## Abstract

Willingness to lay down one’s life for a group of non-kin, well documented historically and ethnographically, represents an evolutionary puzzle. Building on research in social psychology, we develop a mathematical model showing how conditioning cooperation on previous shared experience can allow individually costly pro-group behavior to evolve. The model generates a series of predictions that we then test empirically in a range of special sample populations (including military veterans, college fraternity/sorority members, football fans, martial arts practitioners, and twins). Our empirical results show that sharing painful experiences produces “identity fusion” – a visceral sense of oneness – which in turn can motivate self-sacrifice, including willingness to fight and die for the group. Practically, our account of how shared dysphoric experiences produce identity fusion helps us better understand such pressing social issues as suicide terrorism, holy wars, sectarian violence, gang-related violence, and other forms of intergroup conflict.

Across the historical and ethnographic records, from warriors and soldiers to suicide bombers and religious martyrs, humans have proven capable of not just cooperating within groups, but of making extremely costly personal sacrifices for them. While altruism towards kin is well understood evolutionarily^1^,^2^ extreme self-sacrifice for the sake of non-kin still represents a puzzle. Psychologists have offered a range of explanations for how threatening experiences can trigger increased groupishness^3^,^4^,^5^,^6^,^7^,^8^,^9^,^10^, but these do not address willingness to make the ultimate sacrifice in defense of a group. There is little evidence that such sacrifices, including suicide terrorism, are linked to psychopathology^11^. Rather, a growing body of experimental evidence suggest that willingness to fight and die for the group may be motivated by “identity fusion” – a normal (i.e., not psychopathological) form of group alignment in which the boundary between personal and social identity becomes porous, producing a visceral sense of oneness with the group^12^,^13^,^14^,^15^,^16^,^17^. Driven by the conviction that group members share essence with oneself in ways that can transcend even the bonds of kinship, persons strongly fused to a group report willingness to engage in self-sacrifice. The form that self-sacrifice takes may vary widely in different cultures and historical periods but we argue that one of the pathways to extreme pro-group action, whatever culturally distinctive forms it happens to take, is identity fusion. The identity fusion construct builds on a classic theoretical tradition in psychology – social identity theory^18^. Initial construct validation studies found that strongly fused individuals report perceiving shared essential qualities with a group as well as a sense of reciprocal strength^19^. The identity fusion construct is well grounded in theory and has demonstrated high predictive validity across dozens of experiments, cross-sectional surveys, and longitudinal studies with specialist populations as diverse as revolutionary fighters^20^, victims of atrocities^21^, and civilians loyal to their country^22^,^23^. Overall researchers have shown that identity fusion is a cause of extreme cooperation across cultures, in contrast with the less extreme forms of cooperation motivated by identification (alignment with a group category) and ethnic psychology (the acquisition, storage, and deployment of socially learned group identity markers)^22^,^24^.

One explanation for the extreme cooperation caused by identity fusion is that groupmates are perceived as “psychological kin”^15^,^24^, i.e. that the the human brain, while “wired” for sacrificial behavior towards close kin, makes “mistakes” by facilitating pro-group behavior irrespective of genetic relatedness. The impetus for self-sacrifice by group members is often couched in the language of kinship; and empirical studies show that the effects of identity fusion on pro-national outcomes is partially mediated by feelings of family-like ties toward fellow countrymen^21^,^22^,^25^,^26^. Various religious, military, and terrorist organizations attempt to promote self-sacrifice by exploiting these kin-related instincts^27^. Although kin selection represents a powerful driver of many biological phenomena^1^,^2^, the “psychological kin” explanation is not completely satisfactory. First, it is difficult to imagine how biological mechanisms underlying the“psychological kin” phenomenon could evolve given low genetic relatedness in ancestral human groups (and in our closest relatives – chimpanzees)^28^,^29^ and high costs of self-sacrificial behavior. Second, mechanisms for kin detection in humans^15^,^30^ should act against perceiving unrelated persons as close biological relatives. Finally, in a recent survey of participants in the Libyan uprising of 2011, thousands of whom died in combat, frontline fighters were more likely to choose genetically unrelated fellow revolutionaries in preference to family as the group with which they are most fused^20^. Therefore alternatives to the “psychological kin” explanation need to be explored.

Recent psychological research provides preliminary evidence that a powerful cause of identity fusion is sharing experiences, especially dysphoric (painful and frightening) ones, with group members^15^,^20^,^22^,^24^. Dysphoric experiences may become entrenched as self-defining memories that similarly define fellow group members (Fig. 1). This mechanism of group solidarity is inherently more extreme, and powerful, than oft-cited forms of group commitment such as identification^31^, which have been reliably associated with collective euphoria and group performance^32^. Research indicates that when the group is threatened, fused persons override self-preservation concerns to protect the group at any cost^15^.

This proximate explanation for self-sacrifice motivates us to explore the evolutionary implications of conditioning cooperation on shared past experience. We posit that willingness to perform costly acts for the group is a behavioral strategy that evolved in our ancestors to enable success in high-risk collective activities and between-group conflicts. Groups whose members fused together after experiencing shared dysphoria (i.e., events that negatively impact fitness) would be more likely to prevail in subsequent between-group conflicts in spite of their handicap. Ancestral groups that did not fuse when experiencing shared dysphoria would be less likely to survive in between-group competition. In benign conditions, the willingness to sacrifice for the group would be too costly to sustain. As such, identity fusion should be sensitive to cues of shared dysphoria within the group and to threats imposed from outside the group. Our explanation of individually-costly but group-beneficial behavior thus focuses on evolved coalitionary psychology and tribal instincts^33^,^34^ but emphasizes genetic rather than cultural effects and conditional rather than unconditional expression of self-sacrifice. Here we provide systematic and robust modeling and empirical tests for our explanatory framework. First we investigate theoretically whether conditioning cooperation on types of shared experience can evolve by natural selection. On these grounds we then test the predictions of our models empirically, via correlational, quasi-experimental, and experimental studies. Our findings support the hypothesis that shared dysphoric experiences produce identity fusion and this in turn predicts willingness to fight and die for the group.

## Results

### Mathematical models and theoretical predictions

Our models included many of the standard assumptions of theoretical approaches to within-group cooperation in evolutionary biology. We treated individual willingness to cooperate with group-mates as a genetically controlled trait^1^,^2^. Individual fitness was determined by an outcome of a collective goods game^35^ that the group members participate in. Collective action of group members can be thwarted by free-riding^36^; this problem can be solved to some extent by kin selection, reciprocity, punishment, or group selection^37^,^38^,^39^. Here we offer a novel solution – conditioning cooperation on shared prior experience. In our model, some groups facing a collective action previously had fitness enhancing experiences while others had fitness decreasing experiences. Below we show that conditioning individual efforts in a collective action on these qualities of previously shared experience can evolve by natural selection and can help to solve the free-riding problem. Our model predicts that groups undergoing fitness-decreasing experiences are more likely to contribute substantially to future collective actions. That is, shared past negative experiences can augment future pro-group behavior increasing the overall fitness of both the group and its individual members. Our results however predict a particular evolved social psychology that biases humans to greatly increase cooperation if their groups go through shared negative experiences.

#### Models

More specifically, we considered a population of individuals living in a large number *G* of groups of constant size *n*. Generations are discrete and non-overlapping. We focused on a single collective action^35^,^40^,^41^ that groups attempt to accomplish. The effort of individual *i* in group *j* towards the group’s success in the collective action was modeled as a nonnegative continuous variable *z*_*ij*_; the total group *j* effort of is 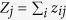. We defined the individual payoff from the collective action as


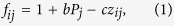


where *b* and *c* are constant benefit and cost parameters. Function *P*_*j*_ = *P*_*j*_(*Z*_*j*_) gives the normalized value of the resource produced by group *j* as a result of collective effort; we normalize *P*_*j*_ relative to a maximum possible reward size (0 ≤ *P*_*j*_ ≤ 1). Relative individual fertility was proportional to 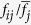, where 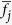 is the average payoff in group *j*.

There are two general types of collective actions in which our ancestors were almost certainly engaged. The first includes group activities such as defense from predators, some types of hunting or food collection, use of fire, etc. The success of a particular group in these activities largely does not depend on the actions of neighboring groups. We will refer to such collective actions as “us vs. nature” contests and define the relative success as


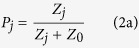


refs 41,42. Here *Z*_0_ is a “half-saturation” constant; the larger *Z*_0_, the more group effort *Z*_*j*_ is needed for the success. The second type of collective action, which we will refer to as “us vs. them” contests, includes direct conflicts and/or competition with other groups over territory and other resources such as mating. The success of one group in an “us vs. them” contest means failure or reduced success for other groups. In these contests, we defined the relative success as


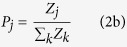


where the sum is over all groups^43^,^44^. We studied “us vs. nature” and “us vs. them” contests separately. Our formulation implied there was an incentive to free-ride on the efforts of group-mates^36^,^35^. The collective action models introduced above belong to a general class of the Volunteer dilemmas^45^,^46^, where individuals would prefer to free-ride on the effort of their group-mates but if nobody else is willing to do it, it may become advantageous to volunteer in spite of the costs involved. It is important to realize that in our models, individuals make contributions to a collective good not because they are “altruistic” but because this increases their fitness.

We extended the above standard model by assuming that groups differ in previous experience which both contributes to the overall probability of the group’s survival and can potentially condition individual cooperation. Specifically, we posited that a random proportion *π* of the groups previously had an euphoric (that is, payoff-increasing) experience whereas a proportion 1 − *π* had a dysphoric (that is, payoff-decreasing) experience. For example, one can think of an “endowment” (e.g. a forest with fruits, or pigs) that the group has initially but may lose because of some random exogenous effects. The loss of the endowment would then represent a dysphoric event experienced by all group members. The previous experience (specified by an indicator variable *E*_*j*_ = 1 for euphoric groups and to 0 for dysphoric groups) and the relative success *P*_*j*_ in the focal collective action jointly controlled the group’s survival probability which is set to be proportional to


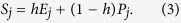


Here 0 ≤ *h* ≤ 1 is a constant parameter measuring the importance of the previous experience for the group’s survival. The equation above uses a simple linear function to specify how previous experience (shared by all group members by assumption) affects group survival. In our model, given everything else the same, the probability of group survival *S* in “euphoric” groups is larger by *h* than that in “dysphoric” groups (*S*_*euphoric*_ = *h* + (1 − *h)P, S*_*disphoric*_ = (1 − *h)P*)). Groups that did not survive were replaced by the offspring of surviving groups (see Methods and the Supplementary Information, SI). In our model, group-level selection favors large individual efforts *z*_*ij*_ (which would increase the probability of group survival *S*_*j*_), while individual-level selection may favor low efforts *z*_*ij*_ (which would reduce the individual costs term *cz*_*ij*_)^47^,^48^.

We assumed that previous experience not only controlled the group’s survival probability, but could also potentially influence individual efforts in the collective action, by triggering different behavioral modules. Specifically we postulated two independent (unlinked) loci with allelic effects *x*_*ij*_ and *y*_*ij*_, the first of which was expressed in individuals with euphoric experience, so that in such individuals *z*_*ij*_ = *x*_*ij*_, and the second was expressed in individuals with dysphoric experience, so that in such individuals *z*_*ij*_ = *y*_*ij*_. In each individual, only one gene was expressed, and all individuals from the same group expressed the same gene. Initially, the allelic effects in both genes in all individuals were very close to zero and the individuals did not contribute much to the collective action. We allowed for mutation, recombination, and migration. We were interested in whether gene effects would increase over time and whether the increase would be similar or asymmetric between the two genes, that is, whether individuals would condition their cooperation on shared past experience.

Our model operated on an evolutionary time-scale focusing on genetic changes leading to the evolution of pro-social behavior conditioned on shared past experience. Individuals’ feelings underlying the development of identity fusion during the individual’s life-time were not modeled explicitly. However, to the extent that identity fusion mediates the relationship between shared past experience and future pro-group actions of an individual (as we show below experimentally), our results also concern the effects of shared experience on identity fusion.

#### Results and predictions

**Figure 2:**
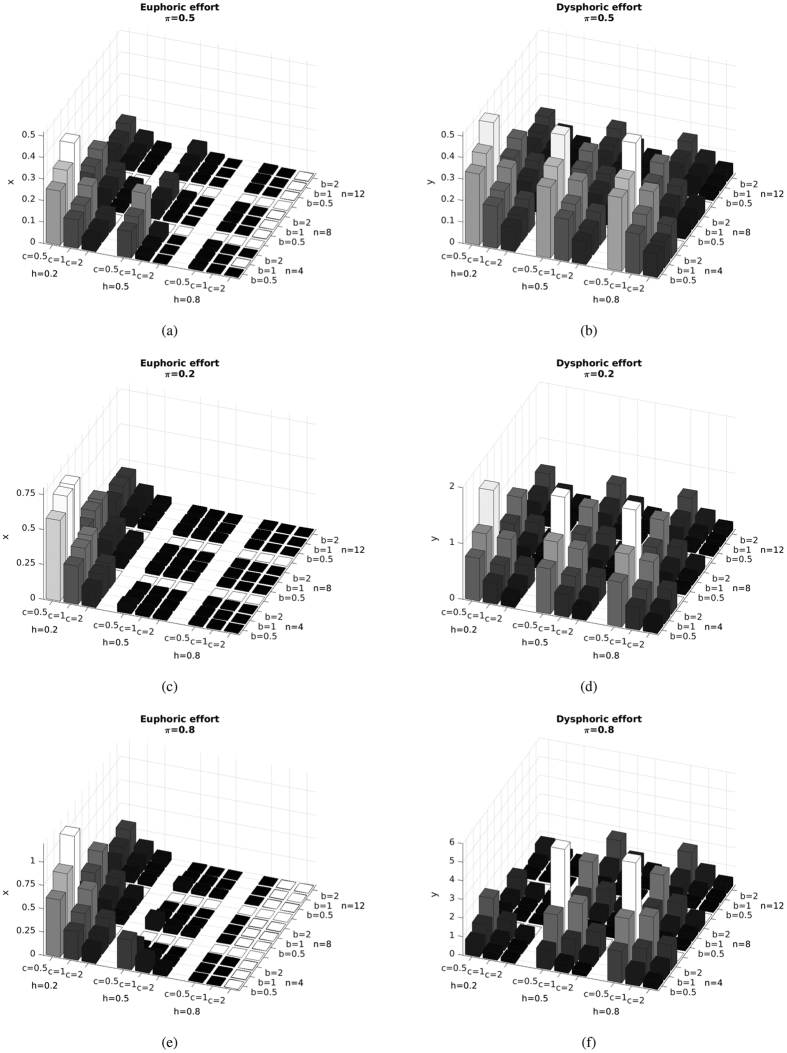
Effects of the benefit *b*, cost *c*, group size *n*, and the weight of previous experience *h* on the average individual efforts in euphoric groups 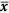 and dysphoric groups 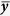. (**a**,**b**) “us vs. nature” contests with the frequency of euphoric groups *π* = 0.5. In these games, the value of *π* does not affect the outcomes. (**c**,**d**) “us vs. them” contests with *π* = 0.2. (**e**,**f**) “us vs. them” contests with *π* = 0.8. The height of the bars is also reflected in their color using the gray colormap (low values in black and high values in white; specific to each individual panel). Notice the difference in the y-scale between subgraphs.

To study our models we used both analytical approximations (shown in the SI) and individual-based simulations. To remove the effects of genetic relatedness, groups were formed randomly at the beginning of each generation. We measured the averages of euphoric 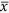 and dysphoric 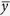 efforts across the whole system at a (stochastic) equilibrium state to which the system evolves. Figure 2 illustrates the effects of five different parameters on the contribution to collective action in the two contests in euphoric and dysphoric groups (see also the SI). Overall, our results lead to the following predictions. First, previous shared experience does affect individual behavior in collective action (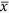 and 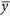 depend on parameters *π* and *h* specifying past experience). Second, dysphoric experience makes individuals contribute more than euphoric experience (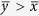). Third, more intense experience results in stronger effects on prosociality (ratio 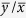 increases with *h*). The intuition behind these results is very simple: groups in poor initial conditions (e.g. with a reduced endowment or fitness) really need to cooperate in order to make it into the future while those in better initial conditions may “afford” somewhat reduced efforts. Fourth, the effect of shared dysphoria on prosocial behavior is much stronger if groups compete directly against other groups (“us vs. them” contests) than if they cooperate against nature (“us vs. nature” contests). Moreover, the effect is stronger in smaller groups (decreasing *n* increases 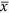 and 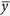). The last two predictions are in line with earlier comparisons of “us vs. them” and ‘us vs. nature” games^41^,^42^. The explanations are that “us vs. them” games impose stronger selection on the underlying genes than “us vs. nature” games^41^,^42^ and that free-riding is a more effective strategy in larger populations^36^. We expect that the effects of the above factors on identity fusion will parallel those on individual actions captured explicitly by our model.

Our models were designed to study the effects of previous experience in the absence of genetic relatedness between group members. We can contrast our results with those for the case when group members are genetically related but the effects of previous experience are absent. The corresponding “us vs. nature” and “us vs. them” contests were studied previously^41^,^42^. With biologically realistic small values of average genetic relatedness^28^, the values predicted by these results can be significantly smaller than those observed in Fig. 2 (see SI). Of course, we do not know realistic values of some important parameters which control the model’s predictions. Nevertheless our results suggest that effects of shared dysphoric experience on willingness to perform individually-costly pro-group acts can potentially be stronger than those of genetic relatedness.

In the models studied above, each individual values the group’s success equally which implies equal degree of identity fusion. In the SI we use results from refs 40,41 to show that highly-fused individuals will exhibit more pro-group sentiments than low-fused individuals. In particular, under conditions of strong between-group competition the model predicts that the efforts of highly-fused individuals will be so high that their fitness will be almost zero. That is, highly-fused individuals are predicted to effectively sacrifice themselves for their groups. Reference 15 provides complementary experimental evidence on the willingness of such individuals to self-sacrifice for their groups.

### Empirical tests

Our models make general predictions concerning cooperation in collective actions. Next, we test five specific predictions focusing on a particularly interesting and extreme type of cooperation - willingness to self-sacrifice for the group. Because we are interested in general behavioral predispositions, we chose a diverse set of samples in eight studies totaling 2,836 individual participants, including citizens of countries, fans of football teams, military veterans, college fraternity/sorority members, martial arts practitioners, and both monozygotic and dyzygotic twins. We ran a total of eight studies, employing correlational (Studies 1, 2, 4, 5, 6), quasi-experimental (Studies 1, 3, 8), and experimental (Study 8) methodologies.

#### Hypothesis 1: Shared experience promotes willingness to preform extreme pro-group action

We ran two studies to test this hypothesis^15^. Both studies distinguish everyday experiences from self-defining experiences (i.e., those that are vividly remembered and are central to one’s self-concept)^49^. In Study 1, American participants were more willing to cooperate (e.g. donate money, volunteer) to solve problems associated with either a natural disaster (*N* = 97) or a terrorist attack (*N* = 98) in the United States when they reported sharing more self-defining (*r* = 0.239, *P* = 0.001) and everyday experiences (*r* = 0.187, *P* = 0.009) with fellow Americans. In Study 2, Americans (*N* = 122) were asked about their willingness to endorse extreme, self-sacrificial pro-group actions. We also measured participants’ levels of identity fusion with their country. Shared experiences increased willingness to endorse extreme pro-group behaviors via increasing identity fusion. This held for both self-defining experiences, 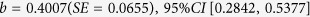, and everyday experiences, 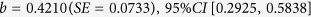.

#### Hypothesis 2: Shared dysphoric experiences more strongly motivate self-sacrifice for the group than euphoric experiences

To test this hypothesis we ran a study on English Premier League football fans (*N* = 725), a collection of modern ‘tribes’ that share dysphoric (e.g. team loss, relegation) and euphoric (e.g. winning cups, embarrassing rivals) experiences^50^. In Study 3, fans of the losing (i.e., dysphoria-producing) teams were more likely to moralize group-related actions (*r* = 0.109, *P* = 0.003) and choose to sacrifice themselves for the sake of an ingroup member in the classic trolley dilemma (*r* = 0.120, *P* = 0.001) than fans of winning (i.e., euphoria-producing) teams. The effects of team support on self-sacrificial responses and pro-group moral endorsements were both mediated by identity fusion, 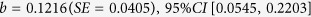 and 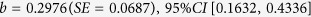, respectively. It is possible that high scores on the trolley dilemma reflect tendencies to self-harm in response to negative affect. Nevertheless, a more plausible explanation is that shared dysphoria motivates extreme cooperation via identity fusion, as high fused individuals of both unsuccessful and successful football teams were found to endorse self-sacrificial behaviour. In earlier studies of group competition involving monetary donations rather than self-reported endorsement of prosocial acts, losing groups increased their contributions while winning groups decreased it^51^,^52^.

#### Hypothesis 3: More intense experiences of shared dysphoria produce stronger effects on self-sacrifice for the group

We ran three studies to test this hypothesis. Military veterans vary widely in exposure to shared dysphoric events^53^,^54^, thus we surveyed U.S. combat veterans of the Vietnam War (*N* = 380) in Study 4. As predicted, greater exposure to shared dysphoric combat experiences (e.g. losing a close co-combatant in battle) predicted both identity fusion (*r* = 0.203, *P* < 0.0001) and willingness to provide support for veterans in need (*r* = 0.184, *P* < 0.0001). Combat experiences increased willingness to provide support to fellow veterans via increasing levels of identity fusion, 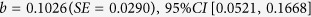. In Study 5, past and current members (*N* = 146) of U.S. college fraternities and sororities who had undergone hazing and other such initiation rituals, were asked about the extent to which the initiation ritual was self-defining^24^. Perceived self-definingness of the experience predicted both identity fusion (*r* = 0.430, *P* < 0.0001) and expressed willingness to sacrifice self for group (*r* = 0.429, *P* < 0.0001). Self-definingness increased pro-group sacrifice by increasing identity fusion, 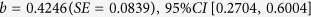. Similarly, in Study 6, we used online advertisements to recruit Brazilian Jiu Jitsu (BJJ) practitioners (*N* = 564), as BJJ promotion events can involve either a painful belt-whipping gauntlet run or less severe practices. This provided an opportunity to compare a population with a significant degree of variation in the dysphoric arousal of important affiliative events, which practitioners are typically unaware of before joining (62.5% reported having “no idea” about their school’s graduation rituals before joining, while a further 16.1% had only “a vague idea”). Despite the significant heterogeneity involved in a worldwide sample, we found that the intensity of belt promotions predicted levels of identity fusion (*ρ* = 0.135, *P* = 0.002), and that identity fusion predicted participants’ stated willingness to risk their lives fighting for the club (*ρ* = 0.542, *P* < 0.0001), as well as their willingness to donate time (*ρ* = 0.508, *P* < 0.0001) and make costly donations of potential prize money (*ρ* = 0.250, *P* < 0.0001) to the club. These relationships remained when controlling for other relevant factors, including age, sex, years training, group identification, and average time training per week. Mediation analyses also showed that elevated intensity of experiences increased participants’ willingness to endorse pro-group behaviors via increasing levels of identity fusion.

#### Hypothesis 4: The effect of shared dysphoria on prosocial behavior is stronger where groups compete directly against other groups, rather than if they cooperate against nature

Study 1 (see above) was designed to test hypothesis 4 as well as hypothesis 1. In Study 1, we found dysphoric contexts involving terrorists elicited more cooperation than those involving natural disasters, *t*(193) = 2.534, *P* = 0.012, Cohen’s *d* = 0.363.

#### Hypothesis 5: The effects of shared dysphoric experience on the willingness to perform pro-group acts can be stronger than those of genetic relatedness

We ran two studies to test this hypothesis. In Study 7, 198 participants either wrote about an experience that has shaped them (Experience), genetically transmitted traits (Genes), or the changing seasons (Control). Participants then imagined interacting with someone who shared the same experience, discovered a long lost sibling, or met a stranger, respectively. Both shared experience (*M* = 32.17, *SD* = 27.21) and shared biology (*M* = 13.66, *SD* = 17.31) increased identity fusion with the person, but shared experiences were a more powerful trigger, *P* < 0.001. Both shared experiences (*M* = 3.19, *SD* = 1.73) and shared genes (*M* = 2.79, *SD* = 1.75) similarly predicted trust for the other person, *P* = 0.173; however, shared genes (*M* = 4.82, *SD* = 2.19) predicted economic sacrifice more than shared experiences (*M* = 4.09, *SD* = 2.11, *P* = 0.045). Identity fusion mediated the relationship between shared experiences/genes and prosocial behavior, *b* = 1.120/0.562 (*SE* = 0.2763/0.1581), 95% *CI* 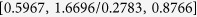. This study partially supports the hypothesis, showing that shared experiences predict levels of identity fusion better than shared genes, and levels of trust as well as shared genes. In Study 8, 260 monozygotic and 246 dizygotic twins^55^ were asked about their shared experiences with their twins, as well as about how fused they were with their twins. Both zygosity (*b* = 0.755 (*SE* = 0.173), 95% *CI* [0.415, 1.094]) and shared experience (*b* = 0.267 (*SE* = 0.033), 95% *CI* [0.202, 0.332]) independently predicted identity fusion. Furthermore, hierarchical regression analyses showed that shared experience continued to predict identity fusion even after controlling for shared genes.

## Discussion

Overall our theoretical and empirical studies both suggest that shared dysphoric experiences are a powerful mechanism for promoting pro-group behaviors which under certain conditions can be extremely costly to the individuals concerned. Our ancestors had a common stake in their group’s fate, especially when facing existential threats. Under threatening conditions, having a shared evolutionary future likely was a more decisive factor in cooperation and self-sacrifice than shared ancestry (i.e., genetic relatedness). A pervasive source of these threats was highly variable environmental conditions during the Late Pleistocene^56^,^57^ making adaptation and survival difficult. Another potential source was competition with other human groups for resources and mating opportunities^58^,^59^,^60^.

Our model captures explicitly how individual efforts in public good games depend on previous group-shared experience. In the model, shared experience has two effects prominent in evolutionary biology and game theory: one on the group survival and another on gene expression. We did not model individual emotions and the sense of identity fusion explicitly. (This would not be possible.) That is, in the triad experience → identity fusion → action, we model explicitly only experience and action. However our experiments as well as earlier work show that identity fusion comes as a “proximate” mediator in the experience → action relationship. Therefore, our results also concern the effects of shared experience on identity fusion.

Previous theoretical research in evolutionary biology has identified a number of mechanisms for the evolution of cooperation^37^,^38^,^39^. Our work brings to light an additional mechanism – conditioning cooperation on shared prior experience. In our models, individuals acquire social instincts to contribute to collective actions because this increases their fitness over evolutionary time. However evolved social instincts may comprise relatively open behaviour programmes that are sensitive to cues such as shared dysphoria, leading to high levels of identity fusion and self-sacrificial acts.

Our proximate explanation for self-sacrifice is that dysphoric experiences and the knowledge that they are shared with the group^61^ shape personal identity and the perception that one’s personal identity is irrevocably tied to the group. The resulting state of identity fusion enables simultaneous activation of group and personal identity. In this light, threats to the group are experienced as threats to self and the drive to defend the group is consequently a form of self-defense^24^. Our empirical findings across study groups suggest a consistently robust trend for dysphoria’s role in extreme cooperation, beyond the effects of group performance or kinship on cooperation that have previously been documented.

There has been recent interest in theoretical literature in the effects of variable environment on the evolution of cooperation^62^,^63^,^64^ with some studies arguing that populations evolving under harsh environments would become more cooperative. Our models are very different in that we consider individual efforts as conditioned on previous group experience. Nevertheless there are some parallels in conclusions: we predict that experiencing an instance of a harsh environment would trigger more cooperative behavior.

Our modeling results naturally have a number of limitations. For example, to isolate the effects of previous experience, we purposely neglected genetic relatedness by randomly forming groups each generation. To simplify analysis, we assumed a simple genetic mechanism underlying instinctive behavior in collective actions while neglecting cultural effects (and transfer of experience between generations). Studying interactions between identity fusion, genetic relatedness, and cultural transmission of behaviors will be an important next step.

Our eight experimental studies provide preliminary empirical evidence for our model, as do other previous studies on the causes and consequences of identity fusion^20^,^22^,^65^. Our empirical findings across study groups suggest a consistently robust trend for dysphoria’s role in extreme cooperation, beyond the effects of group performance or kinship on cooperation that have previously been documented. However, more experimental and longitudinal research is required to substantiate the causal claims made by the model. It is also necessary to develop experiments directly contrasting the hypotheses advanced here with alternative explanations. Furthermore, our studies have all relied on self-report measures. This is in part because the behavioral variables in which we are primarily interested – costly self-sacrificial behaviors – are difficult to measure directly. The use of more benign and commonplace behavioral measures (e.g., economic games) do not approximate our interests, and are therefore poor proxies. Nevertheless, measuring extreme sacrifice directly is impractical and unethical for obvious reasons. Instead we adopt a variety of plausible proxies for extreme sacrifice including identity fusion which has been shown repeatedly to motivate endorsement of extreme sacrifice (e.g. using trolley problems) as well as actual self-sacrifice in real-world correlational studies (e.g. among insurgent groups in Libya^20^). Further field-based experiments, in which we can set up realistic scenarios for costly sacrifice, are required. Finally, we have not tested all the predictions of the model. For example, an intuitive prediction of our model, which we have not directly tested here, is that identity fusion will be stronger in small groups than in large groups. This is consistent with the observation that soldiers are more willing to die for each other (their unit comrades) than for abstract group categories or values (e.g., God and country)^66^.

Our models are meant to capture conditions faced by our ancestors tens of thousands years ago. As such they are not directly applicable to modern groups which have much larger sizes and experience different selection regimes. However our argument (which is standard in evolutionary psychology^67^) is that certain “social instincts” in humans that evolved under ancestral conditions can still be expressed under certain conditions (cf. with “spontaneous altruism” observed in experiments where subjects are forced to make decisions quickly^68^).

Understanding the causes of self-sacrifice for a group is a high priority not only for the evolutionary and psychological sciences but also for society at large. The spirit of self-sacrifice for the group has been a driving force of many historical events^69^,^70^. Many of the world’s ongoing violent conflicts are fuelled by extreme commitment to groups. Nevertheless, people show variation in the extent of fusion with their groups^15^. This heterogeneity could be caused by differences in life history, cultural environment, or developmental factors. Certain groups have high levels of identity fusion, and certain events and/or experiences can cause higher identity fusion that can be exploited to mobilize extreme pro-group behaviors. Understanding altruistic and cooperative behavior by individuals and groups is notoriously difficult as there are multiple forces and factors underlying it, including kinship, reciprocity, punishment, mutualism, and various cultural beliefs and biases. However if we are to address such pressing social issues as suicide terrorism, holy wars, gangland violence, and other forms of intergroup conflict, we should take into account psychological predispositions conditioning extreme cooperation on shared past experiences.

## Methods

### Numerical simulations

We treated individuals as sexual haploid. To implement selection, we used the two-level Fisher-Wright framework^40^,^42^,^71^. Specifically, group selection is captured by making each group in the new generation to independently descend from a group in the previous generation with probability proportional to *S*_*j*_. Individual selection within each group is implemented by first independently choosing 2*n* parents from the group members with probabilities proportional to payoffs *f*_*ij*_ and then producing *n* offspring assuming free recombination. [Results with completely linked genes are qualitatively similar]. Offspring production was followed by random mutation and then by random dispersal of *nG* offspring among *G* groups.

In numerical simulations we considered all possible combinations of the following parameters: benefit of collective action *b* = 0.5, 1.0, 2.0; cost of collective action *c* = 0.5, 1.0, 2.0; group size *n* = 4, 8, 12 (refs 52,53); relative importance of previous experience *h* = 0.2, 0.5, 0.8, and the proportion of groups with dysphoric experience *π* = 0.2, 0.5, 0.8. Parameters that did not change are: number of groups *G* = 1000, mutation rate *μ* = 0.0001, and the standard deviation of mutational effects *σ* = 0.5. To simplify the comparison of the two games we set the half-saturation parameter *Z*_0_ = 1 and made the total contested benefit in “us vs. them” games equal to *bG*, so that that the expected benefit per group is *b* as in “us vs. nature” games. We ran simulations for 20,000 generations 10 times for each combination of parameters (see the SI for more details).

### Experiments

Individual study methodologies, including scale items, as well as individual study data analyses are detailed in the SI. All studies involving human participants were conducted in accordance with APA guidelines and regulations for conducting psychological research. In addition, methods and experimental protocols were approved by the University of Oxford’s Central University Research Ethics Committee, the Murcia University Ethical Committee, or UNSW Human Research Ethics Advisory Panel C. Informed consent was obtained from all participants.

## Additional Information

**How to cite this article:** Whitehouse, H. *et al*. The evolution of extreme cooperation via shared dysphoric experiences. *Sci. Rep.* **7**, 44292; doi: 10.1038/srep44292 (2017).

**Publisher's note:** Springer Nature remains neutral with regard to jurisdictional claims in published maps and institutional affiliations.

## Supplementary Material

Supplementary Information

## Figures and Tables

**Figure 1:**
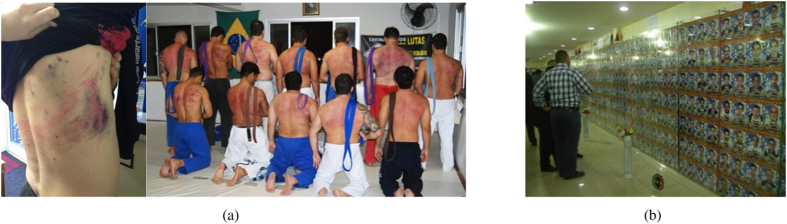
Shared dysphoric experiences. (**a**) Bruises and welts from Brazilian Jiu Jitsu belt whipping gauntlets (Photos: Guillaume Huni). (**b**) Memorial in Misrata to the thousands of revolutionaries in Libya who laid down their lives in the 2011 uprising (Photo: Harvey Whitehouse).

## References

[b1] Hamilton W. D. The genetical evolution of social behaviour I. Journal of Theoretical Biology 7, 1–16 (1964). 5875341 10.1016/0022-5193(64)90038-4

[b2] Frank S. Foundations of Social Evolution (Princeton University Press, Princeton, 1998).

[b3] Festinger L. A Theory of Cognitive Dissonance (Stanford University Press, Palo Alto, CA, 1957).

[b4] Aronson E. & Mills J. The effect of severity of initiation on liking for a group. Journal of Abnormal and Social Psychology 59, 177–181 (1959). 10.1037/h0042162 14422653

[b5] Greenberg J. . Evidence for terror management theory ii: The effects of mortality salience on reactions to those who threaten or bolster the cultural worldview. Journal of personality and social psychology 58, 308–318 (1990). 10.1037//0022-3514.57.4.681 2795438

[b6] Irons W. Religion as a hard-to-fake sign of commitment. In Nesse R. (ed.) The Evolution of Commitment, 292–309 (Russell Sage Foundation, New York, 2001).

[b7] Navarrete C., Kurzban R., Fessler D. M. & Kirkpatrick L. A. Anxiety and intergroup bias: Terror management or coalitional psychology? Group Processes & Intergroup Relations 7, 370–397 (2004).

[b8] Sosis R. Religious behaviors, badges and bans: Signaling theory and the evolution of religion. In McNamara P. (ed.) Where God and Science Meet: How Brain and Evolutionary Studies Alter Our Understanding of Religion, 61–86 (Praeger Publishers, Westport, CT, 2006).

[b9] Sosis R., Kress H. C. & Boster J. S. Scars for war: evaluating alternative signaling explanations for cross-cultural variance in ritual costs. Evolution and Human Behavior 28, 234–247 (2007).

[b10] Harrington J. R. & Gelfand M. J. Tightness-looseness across the 50 united states. Proceedings of the National Academy of Sciences USA 111, 7990–7995 (2014). 10.1073/pnas.1317937111 PMC4050535 24843116

[b11] McCauley C. & Moskalenko S. Mechanisms of political radicalization: Pathways toward terrorism. Terrorism and Political Violence 20, 415–433 (2008).

[b12] Swann W. B. Jr., Gómez A., Seyle D. C., Morales J. F. & Huici C. Identity fusion: The interplay of personal and social identities in extreme group behavior. Journal of Personality and Social Psychology 96, 995–1011 (2009). 19379032 10.1037/a0013668

[b13] Swann W. B. Jr., Gómez A., Huici C., Francisco Morales J. & Hixon J. G. Identity fusion and self-sacrifice: Arousal as a catalyst of pro-group fighting, dying, and helping behavior. Journal of Personality and Social Psychology 99, 824–841 (2010). 20649370 10.1037/a0020014

[b14] Swann W. B. Jr., Gómez A., Dovidio J. F., Hart S. & Jetten J. Dying and killing for one’s group: Identity fusion moderates responses to intergroup versions of the trolley problem. Psychological Science 21, 1176–1183 (2010). 20622141 10.1177/0956797610376656

[b15] Swann W. B. Jr., Jetten J., Gómez A., Whitehouse H. & Bastian B. When group membership gets personal: A theory of identity fusion. Psychological Review 119, 441–456 (2012). 22642548 10.1037/a0028589

[b16] Yoo J., Swann W. B. & Kim K. The influence of identity fusion on patriotic consumption: A cross-cultural comparison of Korea and the US. The Korean Journal of Advertising 25, 81–106 (2014).

[b17] Fredman L. . Identity fusion, extreme pro-group behavior, and the path to defusion. Social and Personality Psychology Compass (2015).

[b18] Tajfel H. & Turner J. C. The social identity theory of intergroup behavior. In Worchel S. & Austin W. G. (eds) Psychology of intergroup relations. 2nd edition, 33–48 (Nelson-Hall, Chicago, IL, 1985).

[b19] Gómez A., Buhrmester M. D., Vázquez A., Jetten J. & Swann W. B. Jr. On the nature of identity fusion: Insights into the construct and a new measure. Journal of Personality and Social Psychology 100, 918–933 (2001). 10.1037/a0022642 21355659

[b20] Whitehouse H., McQuinn B., Buhrmester M. & Swann W. B. Jr. Brothers in arms: Libyan revolutionaries bond like family. Proceedings of the National Academy of Sciences USA 111, 17783–17785 (2014). 10.1073/pnas.1416284111 PMC4273349 25385591

[b21] Buhrmester M., Fraser W., Lanman J., Whitehouse H. & Swann W. J. When terror hits home: fused Americans who saw Boston bombing victims as “family” provided aid. Self & Identity 1–18 (2014).

[b22] Swann W. B. Jr. . What makes a group worth dying for? Identity fusion fosters perception of familial ties, promoting self-sacrifice. Journal of Personality and Social Psychology 106, 912–926 (2014). 24841096 10.1037/a0036089

[b23] Swann W. B. Jr. . Contemplating the ultimate sacrifice: identity fusion channels pro-group affect, cognition, and moral decision making. Journal of Personality and Social Psychology 106, 713–727 (2014). 24749820 10.1037/a0035809

[b24] Whitehouse H. & Lanman J. The ties that bind us: ritual, fusion, and identification. Current Anthropology 6 (2014).

[b25] Atran S., Sheikh H. & Gomez A. For cause and comrade: Devoted actors and willingness to fight. Cliodynamics: The Journal of Quantitative History and Cultural Evolution 5 (2014).

[b26] Vázquez A., Gómez A., Ordon¨ ana J. R. & Paredes B. From interpersonal to extended fusion: relationships between fusion with siblings and fusion with the country. Revista de Psicología Social 30, 1–19 (2015).

[b27] Qirko H. N. Induced altruism in religious, military, and terrorist organizations. Cross-Cultural Research 47, 131–161 (2013).

[b28] Langergraber K. . Genetic differentiation and the evolution of cooperation in chimpanzees and humans. Proceedings of the Royal Society London B 278, 2546–2552 (2011). 10.1098/rspb.2010.2592 PMC3125631 21247955

[b29] Walker R. S. Amazonian horticulturalists live in larger, more related groups than hunter-gatherers. Evolution and Human Behavior 35, 384–388 (2014).

[b30] Lieberman D., Tooby J. & Cosmides L. The architecture of human kin detection. Nature 445, 727–731 (2007). 17301784 10.1038/nature05510 PMC3581061

[b31] Spoor J. R. & Kelly J. R. The evolutionary significance of affect in groups: Communication and group bonding. Group Processes & Intergroup Relations 7, 398–412 (2004).

[b32] Mullen B. & Cooper C. The relation between group cohesiveness and performance: An integration. Psychological Bulletin 115, 210–227 (1994).

[b33] Richerson P. J. & Boyd R. The evolution of subjective commitment to groups: a tribal instincts hypothesis. In Nesse R. M. (ed.) The Evolution of Commitment, 186–220 (Russale Sage Foundation, New York, 2001).

[b34] Vugt M. V. & Park J. H. The tribal instinct hypothesis: evolution and the social psychology of intergroup relations. In Stürmer S. & Snyder M. (eds) Psychology of Helping: New Directions in Intergroup Prosocial Behavior (Blackwell, London, 2008).

[b35] Sandler T. Collective Action: Theory and Applications (University of Michigan Press, Ann Arbor, 1992).

[b36] Olson M. Logic of collective action: Public goods and the theory of groups (Harvard University Press, Cambridge, MA, 1965).

[b37] Nowak M. Evolutionary dynamics (Harvard University Press, Harvard, 2006).

[b38] McElreath R. & Boyd R. Mathematical models of social evolution. A guide for the perplexed (Chicago University Press, Chicago, 2007).

[b39] Sigmund K. The calculus of selfishness (Princeton University Press, Princeton, NJ, 2010).

[b40] Gavrilets S. & Fortunato L. A solution to the collective action problem in between-group conflict with within-group inequality. Nature Communications 5, article 3526, doi: 10.1038/ncomms4526 (2014). PMC3974216 24667443

[b41] Gavrilets S. Collective action problem in heterogeneous groups. Philosophical Transactions of the Royal Society London B 370, 20150016, doi: 10.1098/rstb.2015.0016 (2015). 10.1098/rstb.2015.0016 PMC4633852 26503689

[b42] Gavrilets S. Collective action and the collaborative brain. Journal of the Royal Society Interface 12, article 20141067, doi: 10.1098/rsif.2014.1067 (2015). 10.1098/rsif.2014.1067 PMC4277098 25551149

[b43] Tullock G. Efficient rent seeking. In Buchanan J. M., Tollison R. D. & Tullock G. (eds) Toward a theory of the rent-seeking society, 97–112 (Texas A & M University, College Station, 1980).

[b44] Konrad K. Strategy and dynamics in contests (Oxford University Press, Oxford, 2009).

[b45] Diekmann A. Volunteer’s dilemma. Journal of Conflict Resolution 29, 605–610 (1985).

[b46] Archetti M. Cooperation as a volunteer’s dilemma and the strategy of conflict in public goods games. Journal of Evolutionary Biology 22, 2192–2200 (2009). 19732256 10.1111/j.1420-9101.2009.01835.x

[b47] Wilson D. S. & Wilson E. O. Rethinking the theoretical foundation of sociobiology. Quarterly Review of Biology 82, 327–348 (2007). 18217526 10.1086/522809

[b48] Okasha S. Evolution and the Levels of Selection (Oxford University Press, Oxford, 2009).

[b49] Singer J. & Blagov P. Self Defining Memory Request & Rating Sheet (Connecticut College, New London, CT, 2002).

[b50] Armstrong G. & Giulianotti R. Entering the Field: New Perspectives in World Football (Oxford University Press, Oxford, 1997).

[b51] Tan J. H. W. & Bolle F. Team competition and public goods game. Economics Letters 96, 133–139 (2007).

[b52] Cárdenas J. C. & Mantilla C. Between-group competition, intra-group cooperation and relative performance. Behavioral Neuroscience (2015). 10.3389/fnbeh.2015.00033 PMC4330795 25741258

[b53] Elder G. & Clipp E. Combat experience, comradeship, and psychological health. In Wilson J., Harel Z. & Kahana B. (eds) Human adaptation to extreme stress: From the Holocaust to Vietnam, 131–156 (Plenum Press, New York, 1988).

[b54] King L., King D., Vickers K., Davison E. & Spiro A. Assessing late-onset stress symptomatology among aging male combat veterans. Aging and Mental Health 11, 175–191 (2007). 17453551 10.1080/13607860600844424

[b55] Ordonana J. R. . The Murcia Twin Registry: A population-based registry of adult multiples in Spain. Twin Research and Human Genetic 16, 302–306 (2013). 10.1017/thg.2012.66 23046559

[b56] Richerson P. J., Bettinger R. L. & Boyd R. Evolution on a restless planet: were environmental variability and environmental change major drivers of human evolution. In Woketits F. M. & Ayala F. J. (eds) Handbook of evolution. Vol. 2. The evolution of living systems, 223–242 (Wiley, 2005).

[b57] Richerson P. J. & Boyd R. Rethinking paleoanthropology: A world queerer than we supposed. In Hatfield G. & Pittman H. (eds) Evolution of Mind, 263–302 (Pennsylvania Museum Conference Series, 2013).

[b58] Keeley L. War before civilization (Oxford University Press, New York, 1996).

[b59] Allen M. & Jones T. (eds) Violence and warfare among hunter-gatherers (Left Coast Press, Walnut Creek, California, 2014).

[b60] Lahr M. M. . Inter-group violence among early Holocene hunter-gatherers of West Turkana, Kenya. Nature 529, 394–398 (2016). 26791728 10.1038/nature16477

[b61] Shteynberg G. Shared attention. Perspectives on Psychological Science 5, 579–590 (2015). 10.1177/1745691615589104 26385997

[b62] Lehmann L., Perrin N. & Rousset F. Population demography and the evolution of helping behaviors. Evolution 60, 1137–1151 (2006). 16892965

[b63] Smaldino P., Newson L., Schank J. & Richerson P. Simulating the evolution of the human family: cooperative breeding increases in harsh environment. PLoS One 8, e80753, doi: 10.1371/journal.pone.0080753 (2013). 24278318 PMC3835414

[b64] De Jaegher K. & Hoyer B. By-product mutualism and the ambiguous effects of harsher environments - a game-theoretic model. Journal of Theoretical Biology 393, 82–97 (2016). 26780649 10.1016/j.jtbi.2015.12.034

[b65] Jong J., Whitehouse H., Kavanagh C. & Lane J. Shared negative experiences lead to identity fusion via personal reflection. PLoS One, doi: 10.1371/journal.pone.0145611 (2015). PMC4689389 26699364

[b66] Atran S. Talking to the enemy: violent extremism, sacred values, and what it means to be human (Allen Lane, London, 2010).

[b67] Tooby J. & Cosmides L. The psychological foundations of culture. In Barkow J., Cosmides L. & Tooby J. (eds) The adapted mind: Evolutionary psychology and the generation of culture, 19–136 (Oxford University Press, New York, 1992).

[b68] Rand D. G., Greene J. D. & Nowak M. A. Spontaneous giving and calculated greed. Nature 489, 427–430 (2012). 22996558 10.1038/nature11467

[b69] Khaldun I. The Muqaddimah: an Introduction to History (Princeton University Press, Princeton, NJ, 1958).

[b70] Turchin P. Historical Dynamics: Why States Rise and Fall (Princeton University Press, Princeton, NJ, 2003).

[b71] Schonmann R. H., Vicente R. & Caticha N. Altruism can proliferate through population viscosity despite high random gene flow. PLoS One 8 (2013). 10.1371/journal.pone.0072043 PMC3747169 23991035

