# Supplementary material for: The evolution of extreme cooperation via shared dysphoric experiences
Source: Sci Rep. 2017 Mar 14;7:44292. doi: 10.1038/srep44292 (PMC5349572; doi:10.1038/srep44292)
Supplement: Supplementary Information [file srep44292-s1.pdf]

Supplementary Materials

"The evolution of extreme cooperation via shared dysphoric experiences"

H. Whitehouse, J. Jong, M. D. Buhrmester, Á. Gómez, B. Bastian, C. M. Kavanagh, M. Newson, M. Matthews, J. A. Lanman, R. McKay, and S. Gavrilets

Contents

1. Additional numerical and analytical results.....2
   (a) Numerical simulations.....2
   (b) Analytical approximations.....5
   (c) Genetic relatedness.....6
   (d) Variation in valuation/identity fusion.....7
2. Details of experiments.....9
   (a) Studies 1 and 2: Shared experiences.....9
   (b) Study 3: Shared dysphoria v. euphoria.....13
   (c) Studies 4 to 6: Dysphoric intensity.....17
   (d) Studies 7 and 8: Shared experience vs. genetic relatedness.....24
3. References.....31

1. Additional numerical and analytical results

In our models, individuals make contributions to the collective good not because they are "altruistic" but because this increases their fitness. Our "us vs. nature" games are similar the Volunteer's dilemma games (1–3). Our "us vs. nature" games correspond to models of between-group contests in economics theory (4).

(a) Additional numerical results. Figures (S1-S2) show our results for all values of π considered.

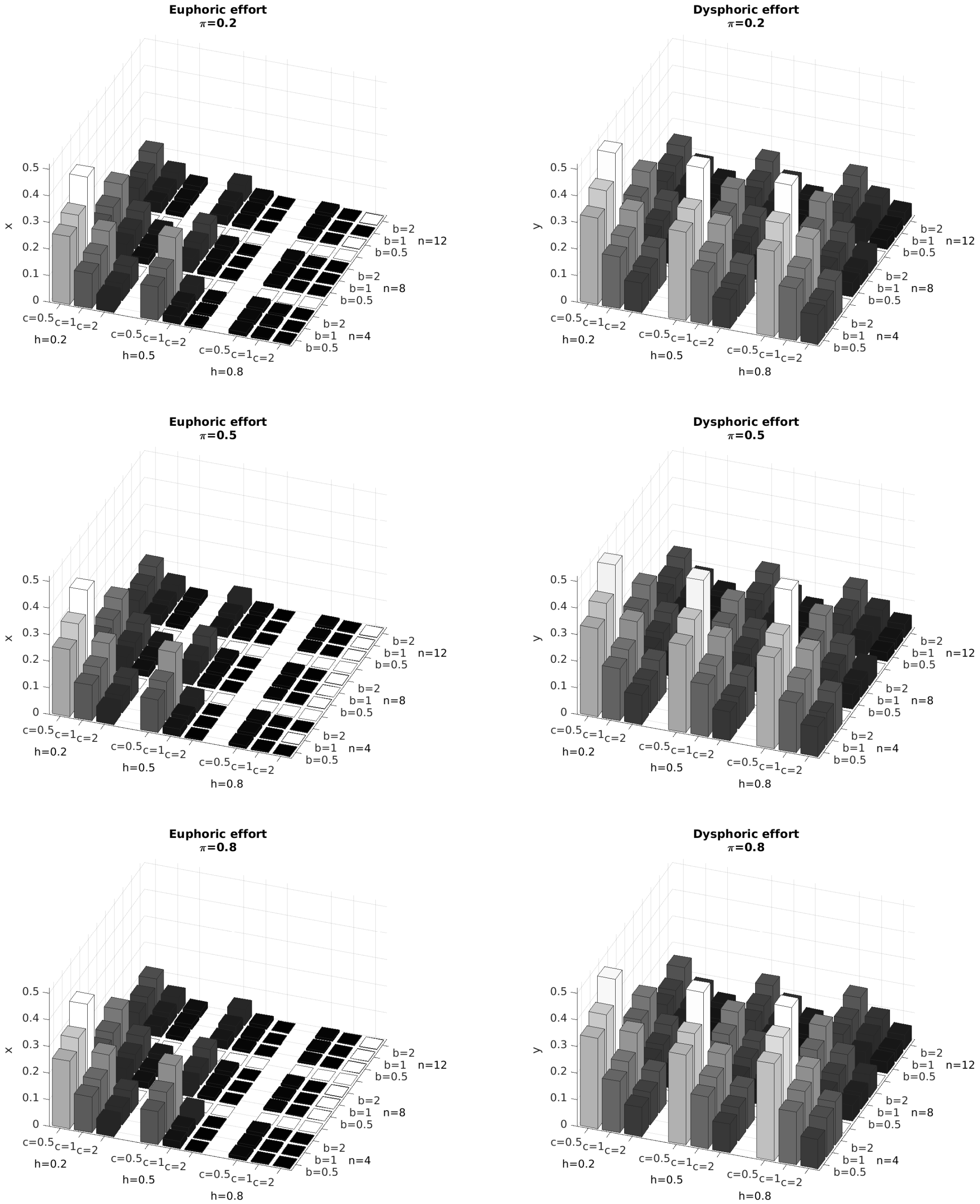

Figure S1: Effects of the benefit b, cost c, group size n, the weight of previous experience h, and the frequency of euphoric groups π on the average individual efforts in euphoric groups $\overline{x}$ and dysphoric groups $\overline{y}$ in "us vs. nature" games. The height of the bars is also reflected in their color using the gray colormap (low values in black and high values in white; specific to each individual panel). Notice the difference in the y-scale between subgraphs.

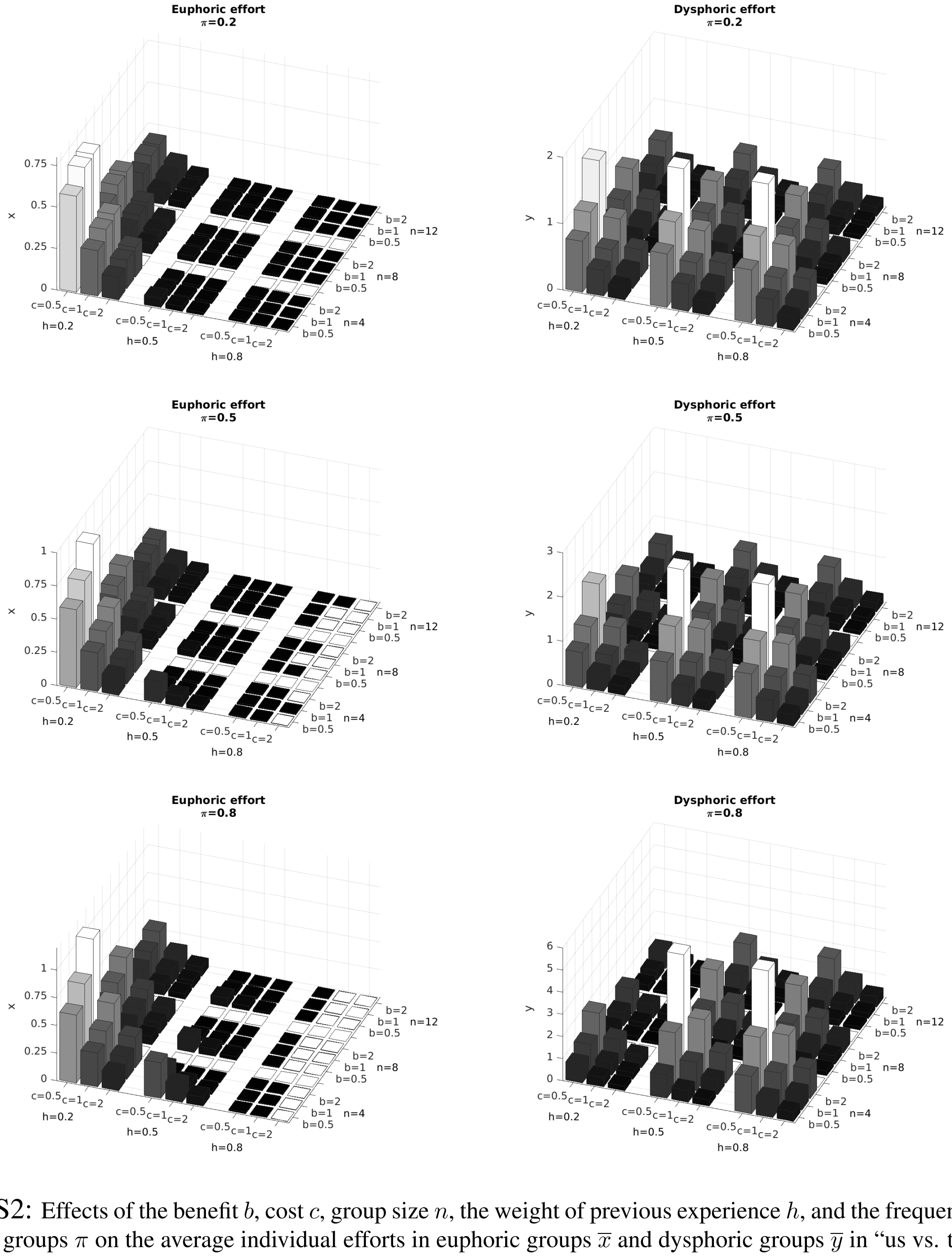

Figure S2: Effects of the benefit b, cost c, group size n, the weight of previous experience h, and the frequency of euphoric groups π on the average individual efforts in euphoric groups $\overline{x}$ and dysphoric groups $\overline{y}$ in "us vs. them" games. The height of the bars is also reflected in their color using the gray colormap (low values in black and high values in white; specific to each individual panel). Notice the difference in the y-scale between subgraphs.

(b) Analytical approximations. We used the invasion analysis and adaptive dynamics methods (5, 6). As any other approximate technique, this approach is based on certain assumptions (5, 6). Therefore it is always important to check analytical approximations against numerical simulations. In our case, the match between predictions and the numerical results reported above is quite satisfactory.

Consider a mutant (u, v) in a resident population (x, y). The mutant can find itself in an euphoric group (with probability π) or dysphoric group (with probability $1-\pi$). The corresponding total group efforts are $X_u = u + (n-1)x, Y_v = v + (n-1)y$, respectively. The mutant's payoffs in an euphoric and dysphoric groups are $f_e = 1 + bP_e - cu$ and $f_d = 1 + bP_d - cv$, where P_e and P_d are the corresponding P values for an euphoric and a dysphoric group with a single mutant. The average payoffs of such groups are $\overline{f_e} = 1 + bP_e - c\,\frac{X_u}{n}$ and $\overline{f_d} = 1 + bP_d - c\,\frac{Y_v}{n}$. The probabilities of survival of an euphoric and a dysphoric group are $S_e = h + (1-h)P_e$ and $S_d = (1-h)P_d$. Then the invasion fitness of mutant (u, v) in a resident population (x, y) is proportional to

$$w(u, v|x, y) = \pi\ S_e\,\frac{f_e}{\overline{f_e}}\ + (1-\pi)\ S_d\,\frac{f_d}{\overline{f_d}}$$

For "us vs. nature" games, the shares of the reward going to a group with a mutant are

$$P_e = \frac{X_u}{X_u + Z_0}, P_d = \frac{Y_v}{Y_v + Z_0},$$

respectively. For "us vs. them" games, these shares are

$$P_e = \frac{X_u}{Z}, P_d = \frac{Y_v}{Z},$$

where $Z = \pi GX + (1-\pi)GY, X = nx, Y = ny$. Dynamic equations for x and y are found by computing appropriate selection gradients (5, 6). Below we present equations for the predicted equilibrium values of x and y.

For "us vs. nature" games, the selection gradients $D_x = \frac{\partial w(u,v|x,y)}{\partial u}\ |_{u=x,v=y}$ and $D_y =$

$\frac{\partial w(u,v|x,y)}{\partial v}\ |_{u=x,v=y}$ are

$$D_{x,nature} = \pi\ \frac{(1-h)[bp(1-p) - cx(n-p) + 1 - p] - h(n-1)c(x+x_0)}{n(x+x_0)(1+bp-cx)},$$
$$D_{y,nature} = (1-\pi)\ \frac{(1-h)[bq(1-q) - cy(n-q) + 1 - q]}{n(y+x_0)(1+bq-cy)},$$

where $p = x/(x+x_0), q = y/(y+x_0)$ and $x_0 = Z_0/n$. The two independent equations $D_{x,nature} = 0, D_{y,nature} = 0$ can be solved for an equilibrium numerically. Note that the frequency of euphoric groups π affects only the rate of evolution but not the equilibrium values.

For "us vs. them" games, assuming the total benefit at stake is bG, the selection gradients are

$$D_{x,them} = \pi\ \frac{(1-h)[bx + (1-ncx)\overline{z}] - (n-1)hc\overline{z}^2}{n\overline{z}[bx + (1-cx)\overline{z}]},$$
$$D_{y,them} = (1-\pi)\ \frac{(1-h)[by + (1-ncy)\overline{z}]}{n\overline{z}[by + (1-cy)\overline{z}]},$$

where $\overline{z} = \pi x + (1-\pi)y$ is the average individual effort in the population. These two coupled equations can be solved analytically for an equilibrium:

$$x^* = \frac{1+b}{nc}\ \frac{(1-h)[1-h-h(b+1-\pi)(1-\frac{1}{n})]}{[1-h-(1-\frac{1}{n})h\pi b][1-h+\pi h(1-\frac{1}{n})]},$$
$$y^* = \frac{1+b}{nc}\ \frac{1-h}{1-h-h\pi b(1-\frac{1}{n})}$$

Under certain conditions, the predicted value of x^* is negative which implies that x decreases to zero. In this case, the equilibrium value of y can be found from equation $[by + (1-ncy)\overline{z}] = 0$ with $\overline{z} = (1-\pi)y$ which results in

$$y^{**} = \frac{1+\frac{b}{1-\pi}}{nc}. \quad (1)$$

Note that increasing π makes y^{**} larger.

(c) Genetic relatedness. To compare the effects of shared experience with those of genetic relatedness we can use results in Ref.(7). Ref. (7) predicts that in the "us vs. nature" contests,

the individual contribution z^* evolves to be positive only if the benefit b is sufficiently large (specifically, $b > cZ_0$). In this case,

$$z^* = z_0 \left(\sqrt{\frac{b}{cZ_0}} - 1 \right) [1 + r(n-1)],$$

where r is the average relatedness within the group and $z_0 = Z_0/n$ (Ref.(7), section 2.1 and Supplementary Material). In the "us vs. them" contests, z^* evolves always to be non-negative and equal to

$$z^* = \frac{1+b}{nc}[1 + r(n-1)]$$

(Ref.(7), section 2.2 and Supplementary Material). Note that the term $1 + r(n-1)$ commonly appears in models of collective action allowing for genetic relatedness (e.g. (7, 8)). For example, let $b = 2, c = 1, n = 8$. With only one sex dispersing as in chimpanzees and likely our ancestors (9), r is predicted (10) to be $1/3(n-1) \approx 0.05$. [This number is close to empirical estimates $r = 0.07$ in (11) and $r = 0.04$ in (12).] Then the two equations above predict $z^* = 0.07$ and $z^* = 0.50$ in "us vs. nature " and "us vs. them" games, respectively. The corresponding numbers from Fig. 1 for dysphoric experience with $h = \pi = 0.5$, are 0.15 and 0.60, respectively. That is, shared dysphoric experience can have effects significantly larger than genetic relatedness.

(d) Variation in fusion. Between-individual variation in fusion can be mathematically captured by introducing variation in how individuals value the group success: a highly-fused individual views the group's success as his/her own success. Now we can use the results in Ref.(13) on collective action in groups with heterogeneity in valuation. In that paper, group members differed in their rank i so that fertility of individual i in group j was defined as

$$f_{ij} = 1 + bP_j n v_i - cz_{ij},$$

where v_i was the share of the group reward going to the individual of rank i or his/her valuation of the resources the groups compete for. If individuals share the reward equally, $v_i = 1/n$.

Ref.(13) showed that only individuals with valuations v_i higher than a certain threshold will make a non-zero effort, while low valuators will free ride, contributing nothing. Individual effort increases with valuation; counter-intuitively however, the individual fertility can decreases with valuation. Under conditions of strong between-group competition, high-rank group members have very low, practically zero, that is, they will act in a self-sacrificial way (e.g. see Figures 4d and 5d in Ref.(13)). Interpreting these results in terms of our model, this means that highly fused individuals (i.e., those with the highest valuation v_i of the group's success) will make the highest effort and can have extremely low fitness.

The behaviour of the highest valuators may seem altruistic but, as explained in (13, 14), actually it is not. Such individuals maximize their fitness by contributing; given the subordinates do not contribute at all, dominants will not be better off by reducing their contribution. Thus, the non-contributors are indeed free-riding, but the contributors are not altruistic; paradoxically, they are acting in their own interest by contributing to the collective good. What is driving their contribution is that they are essentially competing with their counterparts in other groups rather than with their own group-mates.

2. Details of experiments

The experiments were run either online or in person; in each case, ethical approval and informed consent were obtained prior to data collection. In reporting statistical analyses, we followed APA 6th ed. standard statistical abbreviations. E.g., N = sample size, M = mean, SD = standard deviation, SE = standard error of the mean, b = unstandardized regression coefficient, b^* = standardized regression coefficient, 95%CI = confidence interval at 95%, r = Pearson's correlation coefficient, ϱ = Spearman's correlation coefficient, α=Cronbach's α, df = degrees of freedom, also noted in parentheses of test statistics, P = probability value indicating statistical significance.

(a) Studies 1 and 2: Shared experiences

Methods

Participants
American citizens were recruited using Amazon Mechanical Turk (AMT). Participants in Study 1 were paid US$1, and participants in Study 2 were paid US$0.75. There was no overlap in the subjects between Study 1 and Study 2. In Study 1 (N=195), 52.8% of participants were female, 46.2% male, and 1% other; age range was 21 to 71 years (M=37.74, SD=11.25). Demographic data was not obtained for Study 2 to reduce the length of the study; it is reasonable to assume similar demographic representation across both studies (Paolacci and Chandler 2014, Goodman et al. 2013)

Procedure: Study 1
After providing demographic information, participants were introduced to the notion of self-defining experiences. They were given four core characteristics of self-defining experiences. Following Singer and Blagov (2002), a self-defining experience is one that (a) helps explain who you are as an individual and might be an experience you would tell someone else about if you wanted that person to understand you in a profound way; (b) you can remember very clearly and that still feels important to you even as you think about it; (c) can be either positive or negative (or both) in how it makes you feel. The only important aspect is that it leads to strong feelings; (d) that you have thought about many times. Its memory should be familiar to you like a picture you have studied or a song (happy or sad) you have learned by heart.

Participants were then asked three questions about the extent to which they shared self-defining experiences with their fellow Americans:

1. To what extent are your self-defining experiences ones that you had **as an American**.
2. To what extent do you think your **fellow Americans** share similar self-defining experiences with you?
3. To what extent do you think your **fellow Americans** would feel the same way as you do, if they had similar self-defining experiences?

Participants responded to all three questions on a 7-point scale, anchored at 0 (Not at all) and 6 (Very Much). Then, participants answered two questions about "the experiences [they] have in [their] everyday life", using the same 7-point scale:

1. To what extent do you think your **fellow Americans** share similar everyday experiences with you?

2. To what extent do you think your **fellow Americans** would feel the same way as you do, if they had similar everyday experiences?

In the next section participants were either given a scenario in which the United States had just suffered a major terrorist attack (N=98) or one in which the United States had just suffered a major natural disaster (N=97). In either case, participants are told that "Dozens of people have already been killed, but many more are at risk. The cost of reducing the profound negative environmental impact of the disaster, repairing essential infrastructure, and providing food, shelter, and medical attention to victims is estimated at over US$150 million. If such help is not provided soon, the indirect death toll will increase, and the long term damage will be more serious."

Participants were then told that:

"To help fellow Americans in the face of this disaster, a few efforts have begun:

1. Charities are asking for increased short- and long-term donations.
2. Volunteer organizations are recruiting short- and long-term volunteers to help in multiple areas (e.g., administrative, communications, medical assistance, physical labor).
3. Joint publicity campaigns have been launched to raise awareness, funds, and volunteers around the US.
4. Efforts are being made to enact a temporary tax increase to raise funds for the relief and repair effort.
5. Efforts are being made to propose measures to increase the nation's preparedness for future incidents."

On a scale from 0 (Not at all) to 100 (Definitely), participants rated how likely they were to:

1. Make a short term donation
2. Make a long term donation
3. Volunteer in the short term
4. Volunteer in the long term
5. Help spread awareness about opportunities to help
6. Support a temporary tax increase
7. Support measures to prevent future incidents

Procedure: Study 2

Participants answered the same questions about self-defining and everyday memories as in Study 1. Then, they completed a verbal fusion scale (Gómez, Brooks, Buhrmester, Vazquez, Jetten, & Swann, 2011), on a 6-point scale (Strongly Disagree, Disagree, Disagree Somewhat, Agree Somewhat, Agree, Strongly Agree):

1. I am one with America.
2. I feel immersed in America.
3. I have a deep emotional bond with America.
4. America is me.
5. I'll do for America more than any of the other residents would do.
6. I am strong because of America.
7. I make America strong.

Finally, participants were asked about the extent to which they endorsed a series of extreme pro-group behaviours (Gómez et al., 2011), on the same 6-point scale:

1. I would do anything to protect America.
2. I would sacrifice my life if it saved another American's life.

3. I would sacrifice my life if it gave America status.
4. I would fight someone physically threatening another American.
5. I would fight someone insulting or making fun of America as a whole.
6. I would help others get revenge on someone who insulted America.
7. Hurting other people is acceptable if it means protecting America.

Results: Study 1

Descriptive statistics for shared self-defining experiences, shared everyday experiences, and cooperation are as follows:

Table 1.
Descriptive statistics for shared self-defining experiences, shared everyday experiences, and cooperation

Variable	Skew (SE)	Kurt. (SE)	α	M (SD)
Self-defining experiences	-.242 (.174)	-.225 (.346)	.707	4.605 (1.239)
Everyday experiences	-.248 (.174)	-.471 (.346)	.780	5.069 (1.196)
Cooperation	-.311 (.174)	-.442 (.346)	.831	57.108 (21.265)
Cooperation (Natural)	-.345 (.245)	-.065 (.485)	.770	52.383 (19.032)
Cooperation (Terrorist)	-.467 (.244)	-.573 (.483)	.869	60.895 (22.733)

To test Hypothesis 1, we examined Pearson's correlations between shared experiences and cooperation, as reported in the main text.

We also ran a multiple linear regression, with condition (Natural v. Terrorist), self-defining experiences, everyday experiences, and the interaction between condition and each type of shared experience as independent variables, and cooperation as the dependent variable. This allows us to test Hypothesis 4, and to examine the relative contributions of self-defining versus everyday experiences. We found a main effect of condition, $b = 6.615$ ($SE = 2.938$), 95%CI[.821, 12.410], such that willingness to cooperate was higher for the terrorist attack than for the natural disaster. In the main text, we also report a Student's t-test, showing the same effect. Furthermore, we found that with both self-defining and everyday experiences in the same model, there was only a significant effect of self-defining experiences, $b = 4.144$ ($SE = 1.858$), 95%CI[.478, 7.810]. The effect of shared everyday experiences was no longer statistically significant, $b = -.324$ ($SE = 1.894$), 95%CI[-4.061, 3.412]. There were no significant interactions between condition and shared experiences.

Results: Study 2

Descriptive and inferential statistics for shared self-defining experiences, shared everyday experiences, identity fusion, and endorsement of extreme behaviors are as follows:

Table 2.
Descriptive statistics for shared self-defining experiences, shared everyday experiences, identity fusion, and endorsement of extreme behaviors

Variable	Skew (SE)	Kurt. (SE)	α	M (SD)
Self-defining experiences	-.422 (.195)	.185 (.387)	.766	4.718 (1.282)

Everyday experiences	-.587 (.195)	.718 (.387)	.777	5.003 (1.131)
Identity fusion	-.262 (.195)	-.016 (.387)	.936	3.763 (1.089)
Endorsement of ext. beh.	.736 (.195)	.322 (.387)	.912	3.087 (1.355)

Table 3.
Pearson's correlation matrix for shared self-defining experiences, shared everyday experiences, identity fusion, and endorsement of extreme behaviors

	Self-defining experiences	Everyday experiences	Identity fusion	Endorsement of ext. beh.
Self-defining experiences	-	.478**	.654**	.545**
Everyday experiences	.478**	-	.486**	.279**
Identity fusion	.654**	.486**	-	.688**
Endorsement of ext. beh.	.545**	.279**	.688**	-

** P < .001

As distributions for self-defining experiences, everyday experiences, and endorsement of extreme behaviors were mildly skewed, nonparametric correlations are also reported here:

Table 4.
Spearman's correlation matrix for shared self-defining experiences, shared everyday experiences, identity fusion, and endorsement of extreme behaviors

	Self-defining experiences	Everyday experiences	Identity fusion	Endorsement of ext. beh.
Self-defining experiences	-	.421**	.606**	.528**
Everyday experiences	.421**	-	.478**	.235**
Identity fusion	.606**	.479**	-	.683**
Endorsement of ext. beh.	.528**	.235**	.683**	-

** $P < .001$

To further investigate the psychological mechanisms mediating the effect of shared experiences on progroup behavior, we conducted simple mediation analyses using ordinary least squares path analysis in Hayes's PROCESS macro (Model 4) for SPSS (Hayes, 2013). Bias-corrected bootstrap analyses based on 5,000 bootstrap samples were run; such analyses are very robust against violations of normality assumptions. Separate analyses were run for self-defining and everyday experiences. Fusion was entered as a potential mediator between self-defining experiences and endorsement of extreme behaviours (Table 5) and everyday experiences and endorsement of extreme behaviours (Table 6). The confidence intervals for the indirect effects were entirely above zero for both self-defining experiences and everyday experiences. There was also a direct effect of self-defining experiences on endorsement of extreme behaviors independent on its effect on identity fusion. For

everyday experiences there was no significant direct effect detected, the effect was fully mediated by fusion.

Table 5.
Total, direct, and indirect effects for self-defining experiences

	Effect	SE	95% CI
Total effect	.5764	.0735	.4313, .7216
Direct effect	.1757	.0800	.0176, .3338
Unstandardized indirect effect	.4007	.0655	.2842, .5377
Standardized indirect effect	.3791	.0533	.2835, .4952

Table 6.
Total, direct, and indirect effects for everyday experiences

	Effect	SE	95% CI
Total effect	.3343	.0985	.1396, .5289
Direct effect	-.0867	.0694	-.2238, .0504
Unstandardized indirect effect	.4210	.0733	.2925, .5838
Standardized indirect effect	.3515	.0489	.2556, .4490

As in Study 1, we also examined the effects of shared self-defining and everyday experiences in the same model. First, we regressed self-defining and everyday experiences on identity fusion, and found that both independent variables predicted the dependent variable. The effect of self-defining experiences was b = .465 (SE = .057), 95%CI[.351, .578]; the effect of everyday experiences was b = .216 (SE = .065), 95%CI[.088, .344]. Then, we regressed self-defining and everyday experiences on endorsement of extreme behaviours, and found only a significant effect of self-defining experiences, b = .579 (SE = .083), 95%CI[.416, .743]

(b) Study 3: Shared Dysphoria v. Euphoria

Methods

Participants and Procedures

We used longitudinal data (statto.com, 2014) to estimate dysphoria over time for the UK's top football league (the Premier League), considering percentage of home and away games won, drawn and lost, as well as relegations and total league points. Of 35 teams who had been in the Premier League over the last ten years, we selected teams that were currently in the Premier League (to control current media exposure) and that had played at least one previous season in this league. We then focused on the five most consistently successful/euphoria-producing teams (Manchester United, Chelsea, Arsenal, Liverpool and Manchester City) and the five most consistently unsuccessful/dysphoria-producing teams (West Bromwich Albion, Norwich, Sunderland, Hull, and Crystal Palace). An online questionnaire (N=*752*) was advertised to a diverse cross-section of football fans through social media, online fan forum groups, dedicated fan blogs and across student networks. This methodology reflects the diversity of our target sample population, as teams from across England were included. Recent episodes of dysphoria / euphoria were controlled as the study was released for a brief period mid-season and before any significant or decisive matches had taken place. In analyses below, we coded participants who affiliated with one of the five most consistently unsuccessful/dysphoria producing teams vs. one of

the five most successful/euphoria producing teams as a dichotomous variable (euphoria vs. dysphoria club affiliation).

Participants of all teams were given the opportunity to participate to prevent the research purpose being revealed but the relevant teams' fan groups were predominantly contacted to advertise the study. Twenty-seven participants selected a team other than the 10 focal teams of analysis, and we dropped their responses from the dataset, leaving N=725. There was a variation in response rates and we were concerned that our results may have been unduly influenced by the large number of Sunderland fans in the sample (N=290). We therefore re-ran all analyses excluding Sunderland participants and the pattern of results remained consistent. The results we present below include Sunderland fans. Variation in response rates was largely due to the support of a few popular bloggers who were enthusiastic about our research and advertised it to fellow fans following their sites. A £100 prize was offered to all participants as an incentive to complete the study.

Of the 725 participants (M_{age} = 39.5, SD = 15.77), 88.9% were male (11.1% female), 100% had completed secondary level education, and 54.07% had university education. There were null or weak zero-order relationships between educational background, age, gender and outcome variables.

Measures
Identity fusion was assessed using the 7-point verbal scale (*M*=4.28, *SD*=1.23, *α*=.89) (Gómez et al., 2011) in reference to fellow club fans.

Endorsement of self-sacrificial pro-group behaviour was measured with a modified version of an intergroup trolley dilemma (Swann, Gómez, Huici, Morales, & Hizon, 2010) in which participants contemplated sacrificing their lives to save the lives of five fellow club members imperilled on trolley tracks. Participants responded to the question "To what extent would you be willing to sacrifice your life to save the others?" on a 7-point Likert scale (not at all willing to extremely willing).

Moralizing of group-related actions was measured with 4 items on 7-point Likert scales (*α*=.86) as follows:
1. I am obligated to always do right by my club.
2. I feel a sense of duty to my club.
3. If I took advantage of my club, I'd feel immense shame.
4. If I deceived my club in some ways, I would consider myself to be a bad person.

Results

Table 7.
Descriptive statistics for eu- vs dys-phoria, self-sacrifice, moralizing group actions, and identity fusion

Variable	Skew (SE)	Kurt. (SE)	α	M (SD)
Eu- vs. dys-phoria category	-1.142 (.091)	-.697 (.181)	--	.75 (.435)
Self-sacrifice endorsement	.809 (.091)	-.629 (.181)	--	2.640 (1.895)
Moralize group-related actions	-.438 (.091)	-.022 (.181)	.863	4.705 (1.298)

Identity fusion	-.283 (.091)	-.171 (.181)	.936	4.308 (1.224)

Table 8.
Pearson's r correlation matrix for eu- vs dys-phoria, self-sacrifice, moralizing group actions, and identity fusion

	Eu- vs. dys-phoria category	Self-sacrificial endorsement	Moralize group-related actions
Eu- vs dys-phoric category	-		
Self-sacrificial endorsement	.120**	-	
Moralize group-related actions	.109*	.252**	-
Identity fusion	.177**	.174**	.565**

*$P < .01$, ** $P < .001$

Table 9.
Spearman's correlation matrix for eu- vs dys-phoria, self-sacrifice, moralizing group actions, and identity fusion

	Eu- vs. dys-phoric category	Self-sacrificial endorsement	Moralize group-related actions
Eu- vs dys-phoric category	-		
Self-sacrificial endorsement	.101*	-	
Moralize group-related actions	.099*	.240**	-
Identity fusion	.155**	.165**	.541**

*$P < .01$, ** $P < .001$

To test Hypothesis 2, we compared members of dysphoric and euphoric groups by conducting two *t*-tests. In line with Hypothesis 2, we found that compared to euphoric groups, dysphoric groups more strongly endorsed self-sacrifice in the trolley dilemma, $M_{dys} = 2.77$, $SD_{dys} = 1.96$, vs. $M_{eup} = 2.25$, $SD_{eup} = 1.62$, $t\,(723) = 3.24$, $p = .001$, and moralizing group-related actions $M_{dys} = 4.79$, $SD_{dys} = 1.26$, vs. $M_{eup} = 4.46$, $SD_{eup} = 1.37$, $t\,(723) = 2.95$, $p = .003$.

To further investigate the psychological mechanism that mediates the effect of shared dysphoria on progroup behavior, we conducted the same simple mediation analyses in Study 2, using Hayes's (2013) PROCESS macro (Model 4) for SPSS. In the model, the response to the trolley dilemma (i.e., self-sacrifice endorsement) was the outcome, fusion the mediator, and euphoria vs dysphoria club affiliation the predictor. As seen in the Table below, the confidence intervals for the indirect effect, direct effect, and total effect were all above zero.

Table 10.
Total, direct, and indirect effects for self-sacrifice endorsement

	Effect	SE	95% CI
Total effect	.5217	.1603	.2058, .8376
Direct effect	.4001	.1616	.0828, .7174
Unstandardized indirect effect	.1216	.0405	.0545, .2203
Standardized indirect effect	.0279	.0090	.0135, .0500

The same analysis was conducted but with the moralize group-related actions variable as the outcome instead.

Table 11.
Total, direct, and indirect effects moralizing group-related actions

	Effect	SE	95% CI
Total effect	.3252	.1104	.1084, .5419
Direct effect	.0275	.0932	-.1554, .2104
Unstandardized indirect effect	.2976	.0687	.1632, .4336
Standardized indirect effect	.0997	.0225	.0545, .1430

Since relatively few participants were female in the sample, we also conducted the main analyses above without females in the dataset ($N = 645$). Consistent with the above, analyses revealed that compared to euphoric groups, dysphoric groups more strongly endorsed self-sacrifice in the trolley dilemma, $M_{dys} = 2.79$, $SD_{dys} = 1.99$, vs. $M_{eup} = 2.21$, $SD_{eup} = 1.63$, $t\ (643) = 2.73$, $p = .006$, and moralizing group-related actions $M_{dys} = 4.79$, $SD_{dys} = 1.27$, vs. $M_{eup} = 4.46$, $SD_{eup} = 1.37$, $t\ (643) = 3.32$, $p = .001$. Furthermore, the mediation effects were also replicated in the all male subsample. Fusion still mediated the effect on self-sacrifice endorsement, the unstandardized indirect effect $b = .1132$ ($SE = .0405$), 95%CI = [.0497, .2127] and on moralizing group-related actions $b = .2827$ ($SE = .0711$), 95%CI = [.1512, .4298].

(c) Studies 4 to 6: Dysphoric intensity

Study 4
Methods

Participants
The sample consisted of 380 participants (100% male; M_{age}=64.00 years, 89% Caucasian) recruited online via advertisements on the website Facebook. Facebook users could click on an ad with the title "Vietnam Veterans Survey" and be taken to the survey description and informed consent page. All participants indicated at the beginning of the survey that they had served in combat in the Vietnam War as part of the U.S. military.

Procedures
After completing informed consent, participants completed the following scales in this order:

Fusion with fellow Vietnam veterans was measured using the 7-point Likert verbal fusion scale, which was used in Study 2, and adapted for the present target group (Gómez et al., 2011).

Six items measured the extent participants experienced the injury and loss of close others due to combat in Vietnam. Responses were yes (1) or no (0), and summed to produce a total score of shared dysphoric intensity.

1. Did you experience the injury of friends known before the war?
2. Did you experience the loss of friends known before the war?
3. Did you experience the injury of family members?
4. Did you experience the loss of family members?
5. Did you experience the injury of comrades in combat with you?
6. Did you experience the loss of comrades in combat with you?

Three items measured willingness to support veterans in need on 7-point Likert scales:

1. How willing would you be to visit with veterans in need?
2. How willing would you be to volunteer to provide help to veterans in need?
3. How willing would you be to provide support to veterans in need?

Last, participants completed demographic information and were debriefed.

Results
Descriptive and inferential statistics for identity fusion, combat experiences, and willingness to support veterans are as follows:

Table 12.
Descriptive statistics for identity fusion, combat experiences, and willingness to support veterans

Variable	Skew (SE)	Kurt. (SE)	α	M (SD)
Identity fusion	-.848 (.125)	.091 (.250)	.916	5.179 (1.543)
Combat experiences	-.351 (.125)	-.419 (.250)	.578	3.269 (1.446)

Willingness to support	-.664 (.125)	-.582 (.250)	.953	5.085 (1.769)

To test Hypotheses 3, we conducted Pearson's correlations on the extent of combat experiences, identify fusion, and willingness to support veterans. Spearman's correlations are also reported below, in Table 14.

Table 13.
Pearson's correlation matrix for identity fusion, combat experiences, and willingness to support veterans

	Identity fusion	Combat experiences
Identity fusion	-	
Combat experiences	.203**	-
Willingness to support	.435**	.184**

** P < .001

Table 14.
Spearman's correlation matrix for identity fusion, combat experiences, and willingness to support veterans

	Identity fusion	Combat experiences
Identity fusion	-	
Combat experiences	.211**	-
Willingness to support.	.422**	.168**

** P < .001

Similar to Study 2 and 3, simple mediation analyses were conducted using Hayes's PROCESS macro (Model 4) for SPSS. In the model, the sum of combat experiences was the predictor, fusion the mediator, and willingness to support veterans the outcome. As seen in Table 15 below, the confidence intervals for the indirect effect, direct effect, and total effect were all above zero.

Table 15.
Total, direct, and indirect effects

	Effect	SE	95% CI
Total effect	.2357	.0618	.1141, .3572
Direct effect	.1330	.0576	.0198, .2463
Unstandardized indirect effect	.1026	.0290	.0521, .1668
Standardized indirect effect	.0839	.0231	.0431, .1365

Study 5

Methods

Participants

146 past and present U.S. college sorority and fraternity members (52.7% female, 47.3% male; M_{age}=32.45, SD=9.242; Age range=18 to 67 years) were recruited using Amazon Mechanical Turk (AMT), and were paid US$1.

Procedure

Participants completed the 7-item Centrality of Event Scale (Berntsen & Rubin, 2006), on a 5-point scale anchored at 1 (Totally Disagree) and 5 (Totally Agree):

1. I feel that this event has become part of my identity.
2. This event has become a reference point for the way I understand myself and the world
3. I feel that this event has become a central part of my life story.
4. This event has colored the way I think and feel about other experiences.
5. This event permanently changed my life.
6. I often think about the effects this event will have on my future.
7. This event was a turning point in my life.

They then completed the verbal fusion scale (Gomez et al., 2011; see also Study 1) on a 7-point scale (Strongly Disagree, Disagree, Disagree Somewhat, Neither Agree nor Disagree, Agree Somewhat, Agree, Strongly Agree), with their fraternity/sorority as the target group, followed by the pro-group sacrifice measure, which they responded to using the same 7-point scale:

1. I would give up a lot of my time for my [fraternity/sorority] (e.g., to volunteer at events, help with recruiting).
2. I would donate a significant sum of money to my [fraternity/sorority] if it needed it.
3. I would publicly advocate for my [fraternity/sorority] against its critics.
4. I would fight someone physically threatening another member of my [fraternity/sorority].
5. I would fight someone insulting or making fun of my [fraternity/sorority].
6. I would help others get revenge on someone who insulted a member of my [fraternity/sorority].
7. Hurting other people is acceptable if it means protecting my [fraternity/sorority].

Results

Descriptive and inferential statistics for centrality of event, identity fusion, and pro-group sacrifice are as follows:

Table 16.

Descriptive statistics for centrality of event, identity fusion, and pro-group sacrifice

Variable	Skew (SE)	Kurt. (SE)	α	M (SD)
Centrality of event	.167 (.201)	-.800 (.399)	.935	2.492 (1.030)
Identity fusion	-.446 (.201)	-.753 (.399)	.960	3.938 (1.599)
Pro-group sacrifice	-.109 (.201)	-.691 (.399)	.898	3.389 (1.354)

As a further test of Hypothesis 3, we examined the correlations among the perceived centrality of shared dysphoric events, identity fusion, and progroup sacrifice. The Pearson's correlations are displayed in Table 17, while nonparametric correlations are displayed in Table 18.

Table 17.
Pearson's correlation matrix for centrality of event, identity fusion, and pro-group sacrifice

	Centrality of event	Identity fusion	Pro-group sacrifice
Centrality of event	-	.430**	.429**
Identity fusion	.430**	-	.796**
Pro-group sacrifice	.429**	.796**	-

** P < .001

As identity fusion scores were mildly skewed and centrality of event scores were mildly kurtotic, nonparametric correlations are also reported here:

Table 18.
Spearman's correlation matrix for centrality of event, identity fusion, and pro-group sacrifice

	Centrality of event	Identity fusion	Pro-group sacrifice
Centrality of event	-	.424**	.450**
Identity fusion	.424**	-	.740**
Pro-group sacrifice	.450**	.740**	-

** P < .001

A simple mediation analysis was also run as in Study 2, 3, and 4 above. The confidence intervals for the indirect effect was entirely above zero, and there was no evidence for a direct effect of centrality of event on pro-group sacrifice independent on its effect on identity fusion.

Table 19.
Total, direct, and indirect effects

	Effect	SE	95% CI
Total effect	.5633	.0989	.3678, .7589
Direct effect	.1387	.0727	-.0051, .2825
Unstandardized indirect effect	.4246	.0839	.2704, .6004
Standardized indirect effect	.3231	.0590	.2047, .4375

Study 6

Brazilian Jiu Jitsu (BJJ) is a grappling based combat sport and martial arts system that developed in Brazil as an offshoot from Judo. Progression through the system is

structured through a graded belt system and although belt promotion practices vary between schools, there is a widespread and controversial dysphoric practice, known as the belt-whipping gauntlet. These gauntlets involve the promoted student walking along a corridor formed by their training partners whilst being whipped repeatedly by their teammates' using untied belts. The gauntlets tend to last only a few minutes but often result in severe bruising and welts for the recipients (see the image below). Crucially, for the current study there is variance in both the presence and intensity of this event between schools and it therefore provides a unique opportunity to test the hypotheses that: 1) dysphoric events with higher intensities result in higher levels of identity fusion and 2) that this predicts a greater willingness to engage in or endorse costly pro-group practices.

Methods

Participants

563[1] BJJ practitioners were initially recruited for a survey hosted on a dedicated website (www.bjjsurveys.com) through advertisements placed on popular English language BJJ blogs, forums and podcasts. From this sample, 295 participants had experience of belt whipping promotions. In the sample, 95.6% (N=538) of respondents were males and 4.4% females (N=25) (M_{age}=31.23, *SD*=7.070) and North Americans accounted for 60.2%, Western Europeans for 15% and the remaining 24.8% were widely dispersed. As sections of the survey were optional sample sizes are reported. The participants were not compensated for participating but had the option to enter a draw to win training equipment and a £20 prize.

Procedure

An online survey was constructed using Qualtrics software and hosted online. This study was a part of a larger survey on BJJ practitioners, which took 25 minutes to complete in total. After a section on the respondents' history in BJJ, participants were asked:

> *How intense would you consider your belt promotion/grading experiences with your current, or most recent, BJJ school?*

Participants responded using a 6-point scale, anchored at 1 (Not Intense at all) and 6 (Extremely Intense). They were then presented with the 7-item verbal fusion scale adapted for the BJJ school (Gómez et a., 2011; see Study 1), to which they responded on a 6-point scale anchored at 1 (Strongly Disagree) and to 6 (Strongly Agree). Following this, three measures of willingness to sacrifice for the respondent's BJJ school were taken. The first, rated on a 6-point scale anchored at 1 (Strongly Disagree) and 6 (Strongly Agree), measured respondents' willingness to give up time for their BJJ school:

> *If my BJJ school really needed me I would be willing to donate my free time to it.*

The second, rated on the same scale, measured respondents' willingness to risk their lives for their BJJ school:

> *If my BJJ school were threatened, I would be willing to risk my life fighting to defend it.*

1 This total excludes 42 responses which did not complete the relevant identity fusion measures. None of the results reported were altered when these respondents were included in analysis.

These two items, taken from Silver and Brewer (1997) were embedded alongside seven other questions (Yuki, 2003) about the respondent's BJJ school to reduce the potential for demand characteristics. The third and final item occurred in the context of a prize draw. Respondents were offered the chance to participate in a prize draw to win some training equipment and a monetary prize of £20. They were informed that five winners would be selected at random and were given the opportunity to donate some, or all, of the prize to their BJJ club anonymously. Using this voluntary donation we obtained information about participants' willingness to sacrifice monetary resources for their BJJ school. Participation in the draw was optional, as it required respondents to provide contact details.

Results

Descriptive statistics for promotion intensity, identity fusion, and outcome measures are as follows:

Table 20.
Descriptive statistics for promotion intensity, identity fusion, willingness to donate time, willingness to risk life and amount of bonus donated to BJJ school

Variable	Skew (SE)	Kurt. (SE)	α	M (SD)
Intensity of Promotion	.227 (.105)	-1.273 (.210)	-	3.01 (1.680)
Identity Fusion	-.144 (.103)	-.341 (.206)	.885	3.85 (1.066)
Donate Time	-1.219 (.103)	-2.011 (.209)	-	5.06 (1.003)
Risk Life	.711 (.103)	-.677 (.206)	-	2.57 (1.598)
Donate Bonus	.744 (.128)	-1.26 (.251)	-	-6.92 (17.530)

First, an independent *t*-test was conducted to assess whether the emotional intensity of grading experiences was higher for individuals who experienced belt whippings during their promotion events. As expected, individuals from schools with belt-whipping gauntlets reported higher level of intensity (*M*=3.32, *SD*=1.552) in promotion events than those from schools without the practice (*M*=2.68, *SD*=1.749), Welch's *t* (535)=-4.395, 95% CI [-.920, -.361], P<.001.

Second, we examined the overall correlation between intensity and identity fusion, using Spearman's ρ due to non-normal distributions, and found a positive correlation (N=537, ρ =.134, P=.002).

Third, we examined the correlations between fusion and the self-reported measures of willingness to donate time (N=561, ρ =.515, P<.001), and willingness to risk life (N=559, ρ =.546, P<.001), as well as the bonus donation measure (N=377, ρ =.250, P<.001). Inter-measure correlations are as follows:

Table 21.
Spearman's correlation matrix for intensity of promotion, identity fusion, willingness to donate time, willingness to risk life and amount of bonus donated

	Intensity	Identity Fusion	Donate Time	Risk Life	Donate Bonus
Intensity	-	.134**	.094*	.173**	.082
Identity Fusion	.134**	-	.515**	.546**	.250**
Donate Time	.094*	.515**	-	.395**	.282**

Risk Life	.173*	.546**	.395**	-	.181**
Donate Bonus	.082	.250**	.282**	.181**	-

* P < .05 ** P < .001

This presents a third positive test of Hypothesis 3, as the intensity of shared dysphoric experiences is significantly correlated with identity fusion, and two of our three outcome variables.

Simple mediation analyses were run, as in Studies 2 through 5 above, to explore whether fusion mediated the effect of intensity on the three outcome measures. Bias-corrected bootstrap analyses based on 5,000 bootstrap samples were run; such analyses are very robust against violations of normality assumptions. For willingness to donate time, the confidence intervals for the indirect effect were entirely above zero, and there was no evidence of a direct effect of intensity of promotion independent of its effect on identity fusion. Hence, the relationship between intensity and willingness to donate time was fully mediated by fusion. For willingness to risk life, the confidence intervals for the indirect effect were entirely above zero; there was also a direct effect of intensity of ritual. However, the indirect effect on the bonus donation measure was only marginally significant.

Table 22.
Total, direct, and indirect effects on willingness to donate time

	Effect	SE	95% CI
Total effect	.0643	.0257	.0138, .1147
Direct effect	.0226	.0228	-.0222, .0675
Unstandardized indirect effect	.0416	.0120	.0181, .0679
Standardized indirect effect	.0699	.0211	.0310, .1136

N=535

Table 23.
Total, direct, and indirect effects on willingness to risk life

	Effect	SE	95% CI
Total effect	.1744	.0405	.0949, .2539
Direct effect	.0994	.0344	.0318, .1670
Unstandardized indirect effect	.0749	.0226	.0305, .1200
Standardized indirect effect	.0790	.0236	.0320, .1246

N=533

Table 24.
Total, direct, and indirect effects on bonus donation measure

	Effect	SE	95% CI
Total effect	.9021	.5500	-.1796, 1.9837
Direct effect	.6290	.5363	-.4257, 1.6837
Unstandardized indirect effect	.2731	.1480	.0160, .6117
Standardized indirect effect	.0260	.0141	.0018, .0586

N=365

Since relatively few participants were female in the sample, we also conducted the main analyses above excluding the 25 females in the dataset (*N* = 538). Consistent with the above, analyses demonstrated that individuals who had experienced dyspho-

ric belt whippings rated their promotion experiences as more intense, *M*= 3.35*, SD* = 1.55, vs. *M*= 2.71*, SD* = 1.76, *t* (511) = -4.410, *p* < .001. Furthermore, the mediation effects were also replicated in the all male subsample. Fusion still mediated the effect on willingness to donate time, the unstandardized indirect effect *b* = .0398 (*SE* = .0129), 95%CI = [.0160, .0660], willingness to risk life *b* = .0728 (*SE* = .0236), 95%CI = [.0280, .1219] and voluntary donation *b* = .2736 (*SE* = .1534), 95%CI = [.0022, .6134].

(d) Studies 7 and 8: Shared experience and genetic relatedness

Study 7
Methods

Participants
198 Americans (115 males, 83 females; M_{age}=47.15; *SD*=9.99) were recruited via AMT, and were paid US$0.50 for completing the study.

Procedure
Participants were first asked to write a paragraph on a topic to which they were randomly allocated. There were three different conditions: In the Experience condition (N=64) they were asked to "*Write about an experience that has shaped the person you are today*", in the Gene condition (N=67) they were asked to "*Write about the kinds of traits that are genetically transmitted*", and in a Control condition (N=63) they were asked to "*Write about the changing seasons*". Participants in the Experience condition were then asked to imagine meeting a person whom they did not know before, but who also had the very same experience. Participants in the Gene condition were asked to imagine meeting a brother/sister that they did not know they had and who they had never met before. Participants in the Control condition were just asked to imagine meeting a person they had never met before. For female participants the person was named Jane and for male participants the person was named John, such that participants were asked to think about gender matched characters.

All participants were then asked to indicate what they thought their relationship might be like with Jane/John. Specifically, we employed a continuous measure of identity fusion (Jiménez et al., 2015). Participants were asked to "*Please indicate your relationship by clicking and dragging the smaller "me" circle to the position that best captures how you would relate to Jane/John".* This measure provides an indicator of fusion: overlap of the two circles with a value between 0 and 100, with a value of 1 when the circles just begin to overlap and 100 when they are completely overlapping.

To measure willingness to make economic sacrifices, participants were then asked to consider the following scenario:

> You find out that Jane/John needs an urgent and life-saving operation that will cost a large sum of money. What would you be most likely to do?

Participants responded to this scenario by indicating how likely they would be to help Jane/John on scale ranging from 1(*I would be most likely to do nothing*) to 10 (*I would be most likely to do whatever it takes, even selling everything I own*).

Next participants were asked to consider a different scenario, to measure the extent to which they trusted Jane/John:

> Imagine for a moment that you had done something that could potentially ruin your reputation and your life. For example, you may have cheated on your partner, stolen a significant amount of money, or lied about your qualifications to get your job. You have decided that for your own mental health you need to tell someone about this, but the only people you would ordinarily have turned to for advice are unavailable. How likely would you be to tell Jane/John.

Participants responded to this scenario by indicating how likely they would be to tell Jane/John on a scale ranging from 1 (*very unlikely*) to 10 (*very likely*).

Finally, in order to determine whether participants were paying sufficient attention to the survey we included a final item, which simply asked participants to move a slider from where it was sitting (0) all the way to the right (100).

Results

Analysis of the attention-screening variable revealed that four participants did not move the slider all the way to the right (i.e., they had a response value of less than 100). They were excluded from further analysis leaving a total of N=194 participants.

As a test of Hypothesis 5, we compared the effects of fusion of the priming condition—shared experience, shared genes, and control—by running an ANOVA with condition predicting identity fusion. The ANOVA was significant, $F(2,191)=36.55$, $P<.001$, $\eta^2=.28$. Fischer's Least Square Differences post-hoc comparison revealed higher levels of fusion in the experience condition (M=32.17, SD=27.21) than in the gene condition ($M=13.66$, $SD=17.31$) and in the control condition ($M=3.73$, $SD=6.84$) (all $P<.001$). Levels of fusion in the gene condition were also significantly higher than in the control condition ($P=.003$).

To examine whether condition had an effect on our measure of economic-sacrifice we ran the same ANOVA. This revealed a significant effect, $F(2,191)=14.52$, $P<.001$, $\eta^2=.13$. Post-hoc comparison showed that participants in the gene condition were more likely to make economic sacrifices for Jane/John ($M=4.82$, $SD=2.19$) than in the experience condition ($M=4.09$, $SD=2.11$), P=.045, and in the control condition ($M=2.89$, $SD=1.84$), P<.001. Likelihood of economic-sacrifice was also higher in the experience condition than in the control condition, $P=.001$.

The same ANOVA revealed that condition had a significant effect on levels of trust, $F(2,191)=8.19$, $P<.001$, $\eta^2=.08$. Post-hoc comparison revealed participants in the experience condition ($M=3.19$, $SD=1.73$) and in the gene condition ($M=2.79$, $SD=1.75$) were more likely to trust Jane/John than in the control condition ($M=2.02$, $SD=1.49$), P=.008. There was no significant difference in trust between the experience and gene conditions, P=.173.

Table 25.
Pearson's correlation matrix for identity fusion, economic sacrifice, trust

	Identity fusion	Economic sacrifice	Trust
Identity fusion	-	.402**	.521**
Economic sacrifice	.402**	-	.514**

Trust	.521**	.514**	-

** $P < .001$

Mediation Analyses

To examine whether identity fusion with Jane/John explained the effect of condition on economic-sacrifice and trust, we conducted mediation analyses. In particular, we focused on the effect of each experimental condition (experience, gene) compared to the control condition in separate analyses (coded: experimental condition=1 and control condition=0). Simple mediation analyses were conducted using ordinary least squares path analysis in Hayes's PROCESS macro (Model 4) for SPSS (Hayes, 2013). Bias-corrected bootstrap analyses based on 5,000 bootstrap samples were run; such analyses are very robust against violations of normality assumptions.

Focusing first on economic-sacrifice, we examined whether fusion mediated the effect of experience vs. control. As seen in Table 26 below, the confidence intervals for the indirect effect was entirely above zero for the effect of condition (experience vs. control) on economic sacrifice. There was no direct effect of condition (experience vs. control).

Table 26.
Total, direct, and indirect effects for condition (experience v. control) on economic sacrifice

	Effect	SE	95% CI
Total effect	1.2049	.3520	.5082, 1.9015
Direct effect	0.0848	.4000	-.7069, .8765
Unstandardized indirect effect	1.1201	.2763	.5976, 1.6696
Standardized indirect effect	0.2722	.0618	.1492, .3914

We next examined whether fusion mediated the effect of gene vs. control on economic sacrifice. As seen in Table 27 below, the confidence intervals for the indirect effect was entirely above zero for the effect of condition (gene vs. control) on economic sacrifice. There was also a direct effect of condition (gene vs. control).

Table 27.
Total, direct, and indirect effects for condition (gene vs. control) on economic sacrifice

	Effect	SE	95% CI
Total effect	1.9320	.3565	1.2267, 2.6373
Direct effect	1.3701	.3550	.6676, 2.0725
Unstandardized indirect effect	0.5619	.1581	.2783, .8766
Standardized indirect effect	0.1257	.0336	.0649, .1918

Focusing next on trust, we examined whether fusion mediated the effect of experience vs. control. As seen in Table 28 below, the confidence intervals for the indirect effect was entirely above zero for the effect of condition (experience vs. control) on trust. There was no direct effect of condition (experience vs. control).

Table 28.
Total, direct, and indirect effects for condition (experience vs. control) on trust

	Effect	SE	95% CI

Total effect	1.1716	.2861	.6055, 1.7378
Direct effect	0.1870	.3199	-.4462, .8202
Unstandardized indirect effect	0.9847	.2163	.5462, 1.4412
Standardized indirect effect	0.2891	.0564	.1712, .3958

We next examined whether fusion mediated the effect of gene vs. control on trust. As seen in Table 29 below, the confidence intervals for the indirect effect was entirely above zero for the effect of condition (gene vs. control) on economic sacrifice. There was no direct effect of condition (gene vs. control).

Table 29.
Total, direct, and indirect effects for condition (gene vs. control) on trust

	Effect	SE	95% CI
Total effect	0.7752	.2852	.2108, 1.3395
Direct effect	0.1325	.2593	-.3806, .6456
Unstandardized indirect effect	0.6427	.1482	.3699, .9508
Standardized indirect effect	0.1937	.0423	.1161, .2783

Ancillary Analyses

Having demonstrated that shared experiences lead to fusion we also examined whether the nature of the shared experience mattered. Specifically, whether it was participants who wrote about dysphoric experiences that were especially likely to feel fused when imagining another person who had shared that experience. Furthermore, according to theories of costly signaling, it is possible that people felt fused with others who shared self-sacrificial experiences, rather than simply dysphoric experiences. To this end, two researchers rated the personal experience essays on three dimensions: “how dysphoric this person’s experience was”, “how euphoric this person’s experience was”, and “Does this person’s experience demonstrate a willingness to self-sacrifice for others?”. Each of these questions were rated on a scale from 1 (not at all) to 10 (very much so).

Ratings for each dimension were moderately to strongly correlated across the two raters (dysphoria: $r(64)=.83$, $P<.001$; euphoria: $r(64)=.58$, $P<.001$; self-sacrifice: $r(64)=.78$, $P<.001$). As such, a mean score was calculated from both ratings and correlated with fusion. This revealed that the extent to which the personal experience was rated as dysphoric was significantly correlated with fusion, $r(64)=.27$, $P=.033$, marginally correlated with economic sacrifice, $r(64)=.21$, $P=.092$, but uncorrelated with trust, $r(64)=.07$, $P=.586$. Ratings for experiences rated as euphoric were marginally and negatively correlated with fusion, $r(64)=-.22$, $P=.075$, and were negatively although non-significantly correlated with economic sacrifice, $r(64)=-.20$, $P=.117$, but were also uncorrelated with trust, $r(64)=-.10$, $P=.455$. Ratings for experiences considered as self-sacrifice did not correlate with any of the dependent variables (fusion: $r(64)=-.11$, $P=.397$; economic sacrifice: $r(64)=.09$, $P=.491$; trust: $r(64)=-.09$, $P=.463$).

These ancillary analyses point to the importance of shared dysphoric experiences in producing fusion. Perhaps somewhat surprisingly, euphoric experiences tended to have the opposite effects.

Discussion

The findings indicate that both shared experiences and shared genes lead to a tendency to feel fused with another person, but that shared experiences appear to be a

more powerful trigger for fusion than shared genes. Nonetheless, both shared experiences and shared genes predict the tendency to make economic sacrifices on behalf of another person and to trust that other person. Moreover, the tendency to feel fused with that person helps to explain this relationship – sharing experiences or genes with other people increases prosocial behavior and trust due to feelings of fusion. Importantly, the evidence suggests that fusion plays a more important role in translating shared experiences into economic sacrifices, than for shared genes. In the case of shared genes, fusion only partially explains prosocial behavior, suggesting that other factors are also playing a role. In the case of trust, however, fusion appears to play an equally important role in translating shared experiences and shared genes into a tendency to trust another individual.

Study 8

Method

Participants

Five hundred and six participants (280 females and 226 males, M_{age}=55.22, *SD*=6.76) participated in this study. The 260 MZ twins and 246 DZ same-sex twins were recruited from the Murcia Twin Registry (MTR; Ordoñana et al., 2013); the MTR accurately determines zygosity via a standard 12-item questionnaire.

Procedure

Participants responded to a brief questionnaire administered by telephone including measures of shared experiences, and fusion with his/her twin. Participants responded to all these questions on an 11-point scale, anchored at 0 (*completely disagree*) and 10 (*completely agree*).

Shared experiences were rated with a single item:

> Through their life, some siblings experience difficult events. To what extent did you share these kinds of experiences with **your twin.**

Fusion with the twin was measured by a 3-items reduced and adapted scale from Gómez et al. (2011), (Cronbach's α=.74):

1. I am one with **my twin**.
2. I´ll do for **my twin** more than any of my other family members would do.
3. **My twin** is stronger because of me".

Results

Descriptive statistics for dizygotic and monozygotic twins respectively are as follows:

Table 30.
Dizygotic Twins. Descriptive statistics for shared experiences and fusion with the twin.

Variable	Skew (SE)	Kurt. (SE)	α	M (SD)
Shared experiences	-1.257 (.155)	.726 (.309)	--	7.810 (2.799)
Fusion with the twin	-.621 (.155)	.151 (.309)	.729	6.957 (2.174)

Table 31.

Monozygotic Twins. Descriptive statistics for shared experiences and fusion with the twin.

Variable	Skew (SE)	Kurt. (SE)	α	M (SD)
Shared experiences	-1.754 (.151)	2.463 (.301)	--	8.480 (2.393)
Fusion with the twin	-.848 (.151)	-.017 (.301)	.737	7.891 (1.917)

To examine the relationship between the two predictor variables—zygosity and shared experiences—a t-test was run, which showed that zygosity predicted shared experiences, $t(504)=2.93$, P=.004. Nevertheless, a linear regression with both terms entered simultaneously showed that both zygosity and shared experiences independently predicted fusion, b=.755 (*SE*=.173) 95% CI [.415, 1.094] and b=.267 (*SE*=.033) 95% CI [.202, .332] respectively.

As a further test of Hypothesis 5—that is, to determine the relative contributions of zygosity and shared experiences—we conducted two successive linear regressions (i.e., a hierarchical regression analysis; see Table 32). In the first regression (Model 1 below), zygosity was the predictor and fusion the outcome. In the second regression, shared experiences was added to the model as a predictor (Model 2). As shown in the table below, Model 2 which includes shared experiences as a predictor explains more variance in the fusion outcome than Model 1 (see R^2 and F statistics). This suggests that the shared experiences variable uniquely predicts fusion beyond the effect of zygosity.

Table 32.
Summary of hierarchical regression analyses for variables predicting fusion with the twin (N=506).

	Model 1		Model 2	
Variable	*b*	*SE b*	*b*	*SE b*
Zygosity	.934	.182	.755	.173
Shared Experiences			.267	.033
ΔR^2		.050		.109
F *for* ΔR^2		26.316**		65.164**

Note: Shared experiences was mean-centered. ΔR^2 = change in R^2. F *for* ΔR = F-test for change in R^2.
**P<.001.

To further examine the relative contributions of zygosity and shared experiences, we conducted another hierarchical regression, but this time shared experiences was entered as the first predictor (see Table 33). Zygosity was added in the second. The results further support the hypothesis that the shared experiences variable is a stronger predictor of fusion than zygosity, as indicated by Model 1's R^2 = .127, the highest R^2 value out of both hierarchical regressions reported in Table 32 and 33.

Table 33.
Summary of hierarchical regression analyses for variables predicting fusion with the twin (N=506).

	Model 1		Model 2	
Variable	*b*	*SE b*	*b*	*SE b*

Shared Experiences	.285	.033	.267	.033
Zygosity			.755	.173
ΔR^2		.127		.032
F for ΔR^2		73.154**		19.055**

Note: Shared experiences was mean-centered.
**P<.001.

.

References and Notes

[1] Diekmann, A. Volunteer's dilemma. *Journal of Conflict Resolution* **29**, 605–610 (1985).

[2] Archetti, M. Cooperation as a volunteer's dilemma and the strategy of conflict in public goods games. *Journal of Evolutionary Biology* **22**, 2192–2200 (2009).

[3] Archetti, M. & Scheuring, I. Coexistence of cooperation and defection in public goods games. *Evolution* **65**, 1140–1148 (2010).

[4] Konrad, K. *Strategy and dynamics in contests* (Oxford University Press, Oxford, 2009).

[5] Geritz, S. A. H., Kisdi, E., Meszéna, G. & Metz, J. A. J. Evolutionary singular strategies

and the adaptive growth and branching of the evolutionary tree. *Evolutionary Ecology* **12**, 35–57 (1998).

[6] Waxman, D. & Gavrilets, S. 20 questions on adaptive dynamics. *Journal of Evolutionary Biology* (2004).

[7] Gavrilets, S. Collective action and the collaborative brain. *Royal Society Interface* **12**, article 20141067 (2015).

[8] Archetti, M. & Scheuring, I. Game theory of public goods in one-shot social dilemmas without assortment. *Journal of Theoretical Biology* **299**, 9–20 (2012).

[9] Chapais, B. *Primeval kinship: how pair-bonding gave birth to human society* (Harvard University Press, Cambridge, 2008).

[10] Taylor, P. D., Irwin, A. J. & Day, T. Inclusive fitness in finite deme-structured and stepping-stone populations. *Selection* **1-3**, 153–163 (2000).

[11] Bowles, S. Did warfare among ancestral hunter-gatherers affect the evolution of human social behaviors? *Science* **324**, 1293–1298 (2009).

[12] Langergraber, K. *et al.* Genetic differentiation and the evolution of cooperation in chimpanzees and humans. *Proceedings of the Royal Society London B* **278**, 2546–2552 (2011).

[13] Gavrilets, S. & Fortunato, L. A solution to the collective action problem in between-group conflict with within-group inequality. *Nature Communications* **5**, article 3526 (doi:10.1038/ncomms4526) (2014).

[14] Gavrilets, S. Collective action problem in heterogeneous groups. *Philosophical Transactions of the Royal Society London B* **370**, 20150016 (2015).

[15] Berntsen, D., & Rubin, D. C. (2006). The centrality of event scale: A measure of integrating a trauma into one's identity and its relation to post-traumatic stress disorder symptoms. *Behaviour Research and Therapy, 44*, 219-231.

[16] Gómez, Á., Brooks, M. L., Buhrmester, M. D., Vázquez, A., Jetten, J. & Swann, W. B. Jr. (2011). On the nature of identity fusion: Insights into the construct and a new measure. *Journal of Personality and Social Psychology, 100*, 918-933.

[17] Goodman, J. K., C. E. Cryder, and A. Cheema. "Data collection in a flat world: The strengths and weaknesses of Mechanical Turk samples." *Journal of Behavioral Decision Making* 26.3 (2013): 213-224

[18] Hayes, A. F. (2013). Introduction to mediation, moderation, and conditional process analysis. New York: The Guilford Press.

[19] Ordoñana, J. R, Rebollo-Mesa, I., Carrillo, E., Colodro-Conde, L., García-Palomo, F. J., González-Javier, F., Sánchez-Romera, J. F., et al. (2013). The Murcia Twin Registry: a population-based registry of adult multiples in Spain. *Twin Research and Human Genetics, 16*, 302-306.

[20] Paolacci, G. and J. Chandler. "Inside the Turk: understanding Mechanical Turk as a participant pool." *Current Directions in Psychological Science* 23.3 (2014): 184-188.

[21] Silver, M., & Brewer, M. B. (1997, May). *Perceptions of multiple group loyalty, and a new scale for loyalty measurement*. Paper presented at the annual meeting of the American Psychological Society, Washington, DC.

[22] Singer, J.A. & Blagov, P. (2002). *Classification system & scoring manual for self-defining memories*. Connecticut College: New London, CT.

[23] Swann, W.B., Jr., Gómez, A., Huici, C., Morales, F., & Hixon, J. G. (2010). Identity fusion and self-sacrifice: Arousal as catalyst of pro-group fighting, dying and helping behavior. *Journal of Personality and Social Psychology, 99*, 824–841.

[24] Yuki, M. (2003). Intergroup comparison versus intragroup relationships: A cross-cultural examination of social identity theory in North American and East Asian cultural contexts. *Social Psychology Quarterly, 66*, 166-183.
